# Supplementary material for: The Arabidopsis endosperm is a temperature-sensing tissue that implements seed thermoinhibition through phyB
Source: Nat Commun. 2023 Mar 7;14:1202. doi: 10.1038/s41467-023-36903-4 (PMC9992654; doi:10.1038/s41467-023-36903-4)
Supplement: Supplementary file 1 — Supplementary Information [file 41467_2023_36903_MOESM1_ESM.pdf]

Supplementary Figure 1

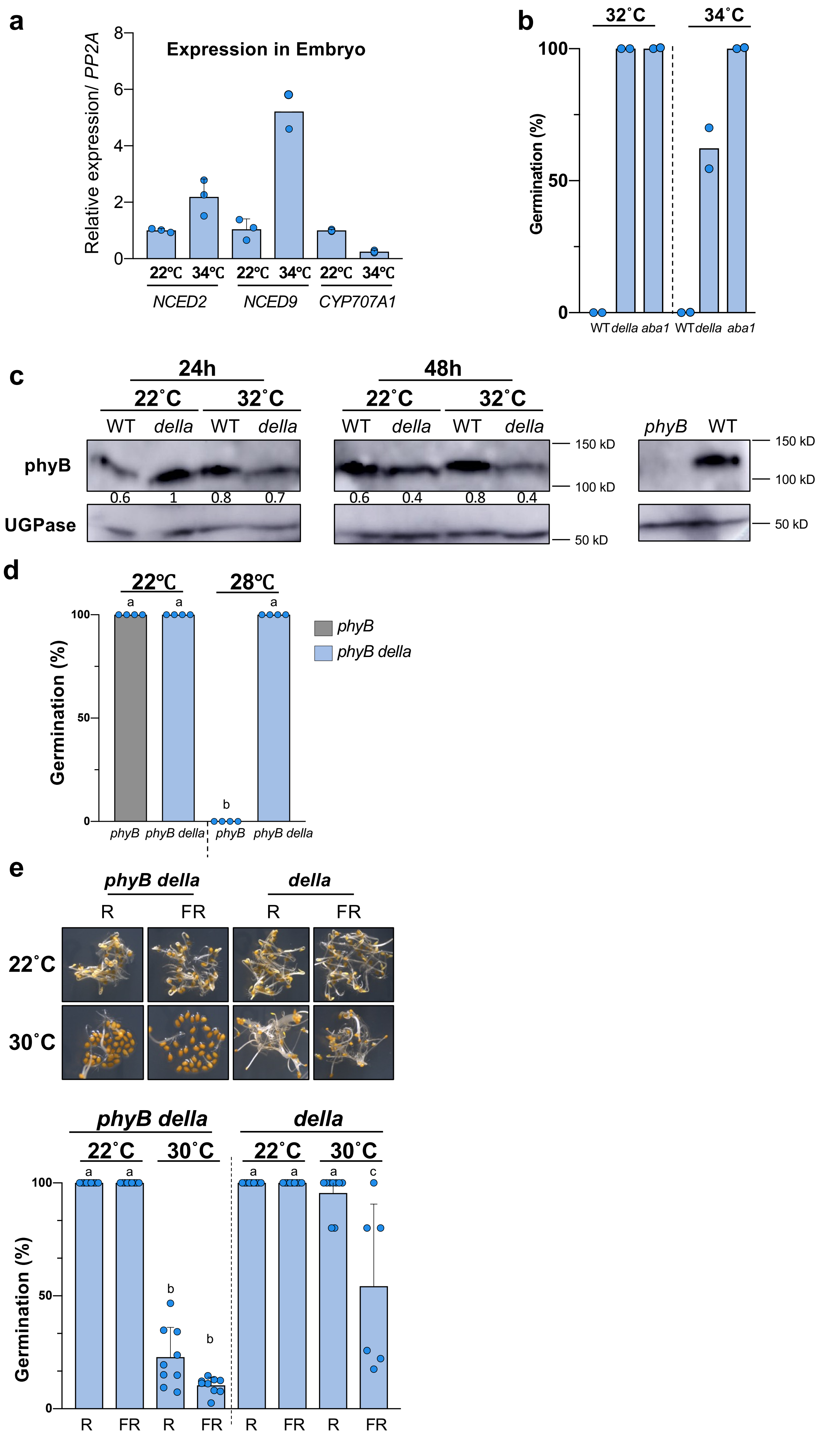

Supplementary Figure 1. DELLA factors promote ABA release from endosperm when phyB signaling is low.

a. Relative *NCED2*, *NCED9* and *CYP707A1* mRNA accumulation in WT embryos dissected from WT seeds after 36h upon seed imbibition under white light (50 μmol/m^2/s) at indicated temperatures. Total RNA was isolated from 100 embryos. Histograms show the average expression value from 3 technical replicates (2 for *NCED9* at 34˚C). Data represent mean standard deviation (+). Expression levels were normalized to those of *PP2A.*

b. Average germination percentage of WT, *della* and *aba1* seeds cultured 3 days under white light (50 μmol/m^2/s) at 32˚C and 34˚C in 2 independent seed batches (n≥50 seeds).

c. PHYB protein levels in WT and *della* seeds cultured under white light (50 μmol/m^2/s) at 22˚C or 32˚C for 24h and 48 h. Numbers represent PHYB levels normalized to those of UGPase. PhyB antibody control: protein extract from WT and *phyB* seedlings grown for 2 days in darkness. This experiment was repeated two times with similar results.

d. Average germination percentage of *phyB* and *phyB della* seeds cultured 3 days under white light (50 μmol/m^2/s) at 22˚C and 28˚C in 4 independent seed batches (n≥50 seeds). Data represent mean standard deviation (+). Statistical treatment and lower-case letters are used as in Figure 1b.

e. Upper panel: representative pictures of *phyB della* and *della* seeds cultured for 3 days in darkness at 22˚C and 30˚C after receiving a 5 minutes far-red light (FR) pulse (3.69 μmol/m^2/s) or a FR pulse followed by a 5 minutes red light (R) pulse (14.92 μmol/m^2/s) 2h upon seed imbibition. Lower panel: average germination percentages after 3 days in 9 independent seed batches (6 for *della* FR 30 ˚C) (n≥40 seeds). Data represent mean standard deviation (+). Statistical treatment and lower-case letters are used as in Figure 1b.

Supplementary Figure 2

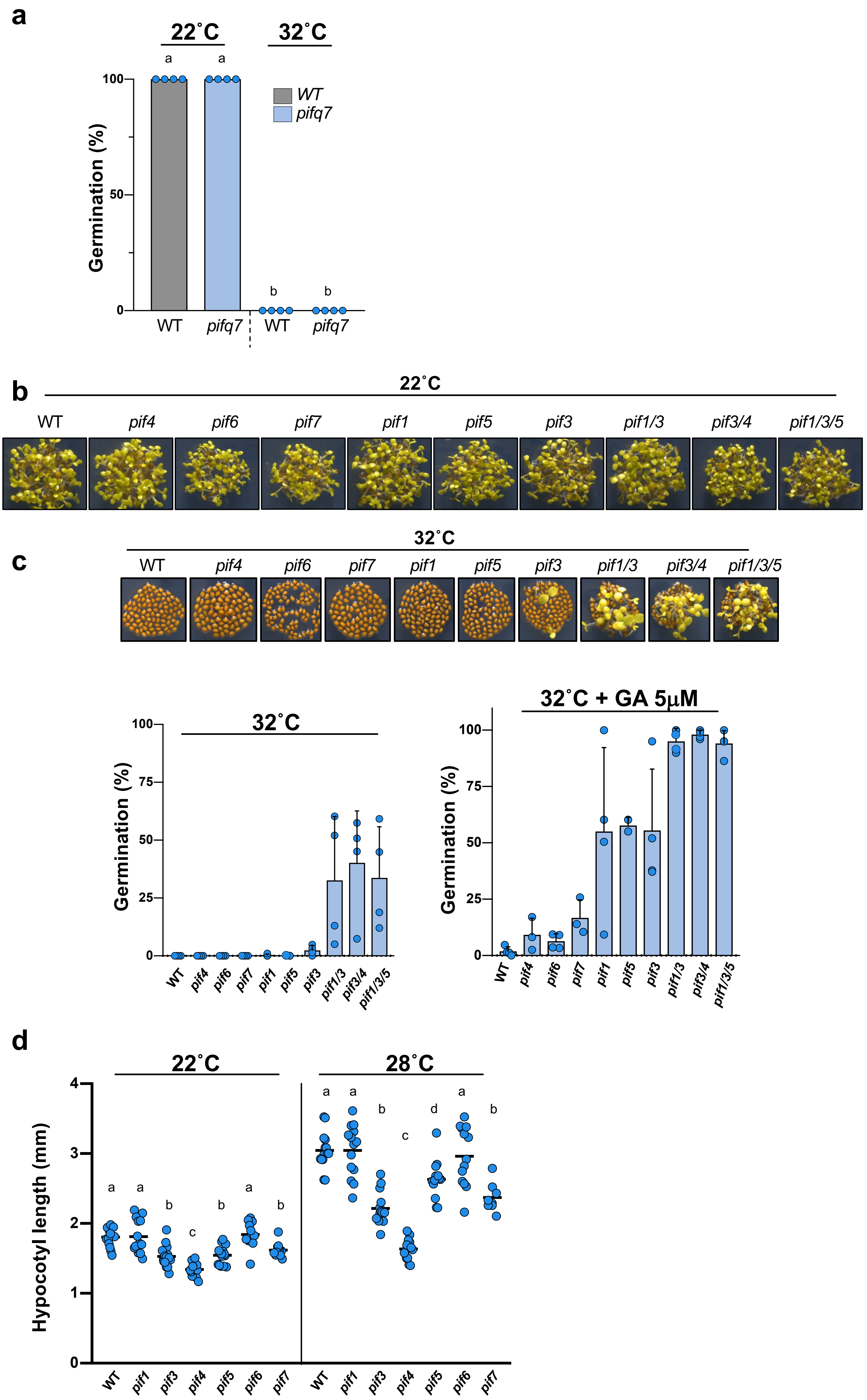

Supplementary Figure 2. Endospermic PIF3 promotes seed thermoinhibition by promoting ABA synthesis and release.

a. Average germination percentage of WT and *pifq7* seeds cultured for 3 days under white light (50 µmol/m^2/s) at 22˚C and 32˚C in 4 independent seed batches (n≥50). Data represent mean standard deviation (+). Statistical treatment and lower-case letters are used as in Figure 1b.

b. Representative pictures of WT and *pif* seeds cultured for 3 days under white light (50 µmol/m^2/s) at 22˚C.

c. Upper panel: representative pictures of WT and *pif* seeds cultured for 6 days under white light (50 µmol/m^2/s) at 32˚C. Average germination percentage of WT and *pif* seeds cultured, under white light (50 µmol/m^2/s), for 6 days at 32˚C (lower left panel) and for 4 days at 32˚C in presence of 5µM GA (lower right panel). Four seed batches were analyzed, except for the GA conditions, where 2 seed batches are used for *pif5* and 3 seed batches were used for *pif4* and *pif7* (n≥50). Data represent mean standard deviation (+).

d. Hypocotyl length (mm) in WT and *pifs* seedlings grown according to Koini et al, 2009: seeds were stratified in darkness for 3 days and transferred under white light (50 µmol/m^2/s) to 22˚C for 4 days and thereupon either kept at 22˚C or transferred at 28˚C for 3 days. N=14 embryos for all genotypes except for *pif6* (22˚C n=13 and 28˚C n=8), *pif7* (22˚C n=12 and 28˚C n=8) and *pif3*, *pif4*, *pif5* at 22˚C (n=15). Data represent individual and mean (horizontal line) hypocotyl length for each genotype. Statistical treatment and lower-case letters are used as in Figure 1b.

Supplementary Figure 3

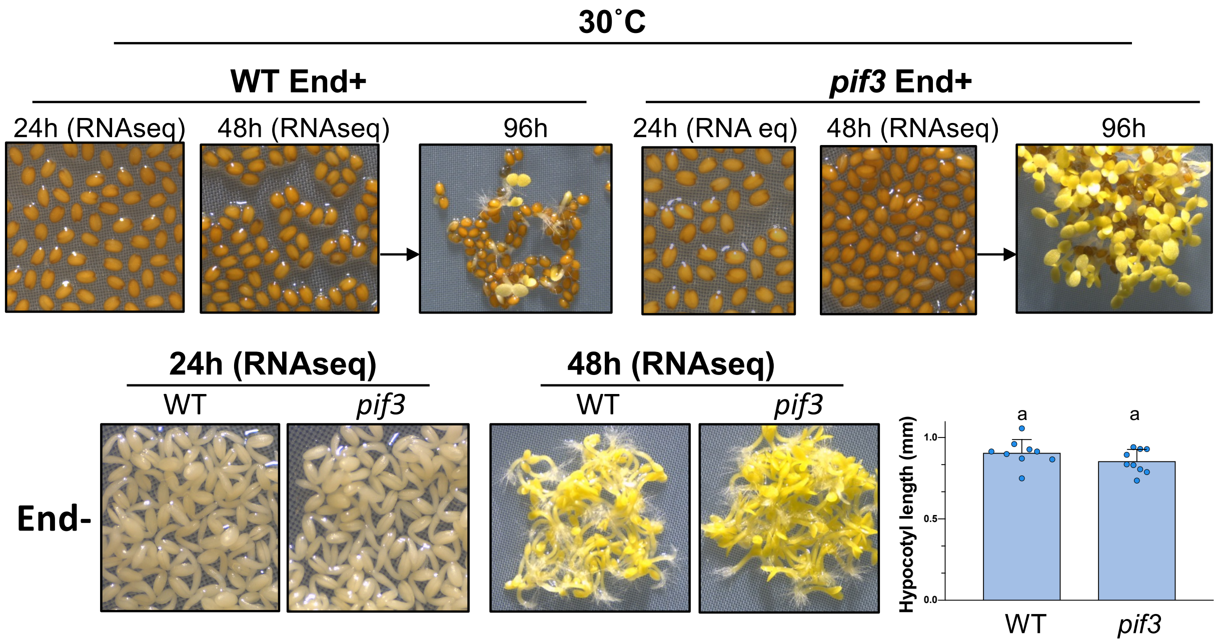

Supplementary Figure 3. Material for RNAseq

Upper panel: pictures show WT and *pif3* seeds 24h, 48h and 96h upon seed imbibition under white light (50 µmol/m^2/s) at 30˚C. Total RNA was isolated from endosperms were dissected at 24h and 48h and used for RNAseq analysis. Lower panel: pictures show WT and *pif3* embryos dissected 4 hours after seed imbibition and incubated for 24h and 48h at 30˚C (End-). Histogram shows hypocotyl length (mm) measured in End- seedlings at 48h (n=9 embryos). Data represent mean standard deviation (+). Statistical treatment and lower-case letters are used as in Figure 1b.

Supplementary Figure 4

a

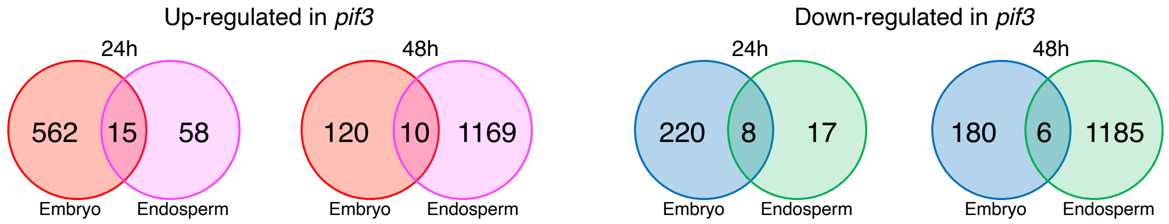

b

Genes up-regulated in *pif3*

GO biological process	P-value	Fold-enrichment
**Embryo at 24h**		
photosynthesis, light harvesting in photosystem I (GO:0009768)	22.33	1.63E-07
photosynthesis, light harvesting (GO:0009765)	12.46	2.65E-05
water transport (GO:0006833)	9.99	1.23E-02
fluid transport (GO:0042044)	9.99	1.23E-02
response to far red light (GO:0010218)	9.14	5.89E-03
plant-type secondary cell wall biogenesis (GO:0009834)	6.47	1.48E-04
photosynthesis, light reaction (GO:0019684)	4.46	4.97E-02
plant-type cell wall biogenesis (GO:0009832)	4.33	2.49E-05
cell wall polysaccharide metabolic process (GO:0010383)	4.28	8.75E-03
cell wall macromolecule metabolic process (GO:0044036)	4.17	1.53E-03
cell wall biogenesis (GO:0042546)	3.62	2.88E-06
response to light intensity (GO:0009642)	3.4	5.74E-04
cell wall organization or biogenesis (GO:0071554)	3.33	3.61E-13
plant-type cell wall organization or biogenesis (GO:0071669)	3.31	7.98E-06
cellular polysaccharide metabolic process (GO:0044264)	3.09	3.35E-02
polysaccharide metabolic process (GO:0005976)	2.85	3.40E-03
plant epidermis development (GO:0090558)	2.82	1.48E-04
root morphogenesis (GO:0010015)	2.62	1.25E-03
carbohydrate metabolic process (GO:0005975)	2.47	1.62E-05
plant organ morphogenesis (GO:1905392)	2.38	1.03E-03
**Endosperm at 24h**		
olefinic compound metabolic process (GO:0120254)	30.9	3.53E-02
flavonoid biosynthetic process (GO:0009813)	23.8	8.92E-03
response to chemical (GO:0042221)	2.51	5.59E-04
response to stimulus (GO:0050896)	1.94	5.09E-04
**Embryo at 48h**		
response to cold (GO:0009409)	5.14	3.81E-02
response to light stimulus (GO:0009416)	2.76	1.03E-02
response to radiation (GO:0009314)	2.7	1.48E-02
response to abiotic stimulus (GO:0009628)	2.2	1.42E-03
response to stimulus (GO:0050896)	1.66	9.70E-04
**Endosperm at 48h**		
defense response by callose deposition in cell wall(GO:0052544)	13.37	9.61E-04
defense response by cell wall thickening (GO:0052482)	12.58	1.42E-03
defense response by callose deposition (GO:0052542)	10.46	3.21E-04
indole glucosinolate metabolic process (GO:0042343)	10.01	2.36E-02
callose deposition in cell wall (GO:0052543)	9.34	8.02E-04
cell wall thickening (GO:0052386)	9.2	2.60E-04
callose localization (GO:0052545)	7.74	1.31E-04
polysaccharide localization (GO:0033037)	7.23	2.64E-04
toxin catabolic process (GO:0009407)	6.2	8.66E-03
nucleotide-sugar biosynthetic process (GO:0009226)	6.08	2.63E-02
nucleotide-sugar metabolic process (GO:0009225)	5.83	6.04E-03
glycosyl compound catabolic process (GO:1901658)	5.38	3.01E-02
toxin metabolic process (GO:0009404)	5.04	1.87E-06
cellular response to hypoxia (GO:0071456)	4.97	8.40E-15
cellular response to decreased oxygen levels (GO:0036294)	4.93	1.13E-14
cellular response to oxygen levels (GO:0071453)	4.91	1.32E-14
response to red light (GO:0010114)	4.85	7.75E-05
carbohydrate derivative catabolic process (GO:1901136)	4.81	8.43E-04
indole-containing compound metabolic process (GO:0042430)	4.31	1.35E-07
response to hypoxia (GO:0001666)	4.27	8.84E-1

Genes down-regulated in *pif3*

GO biological process	P-value	Fold-enrichment
**Embryo at 24h**		
seed oilbody biogenesis (GO:0010344)	88.77	2.31E-06
lipid storage (GO:0019915)	41.32	2.46E-08
response to freezing (GO:0050826)	24.66	1.15E-02
dormancy process (GO:0022611)	22.66	2.29E-05
seed dormancy process (GO:0010162)	22.66	2.29E-05
seed maturation (GO:0010431)	22.44	5.93E-12
maintenance of location (GO:0051235)	17.52	2.81E-06
cold acclimation (GO:0009631)	12.89	3.63E-02
multicellular organismal reproductive process (GO:0048609)	9.84	1.80E-08
multicellular organism reproduction (GO:0032504)	9.59	2.64E-08
anatomical structure maturation (GO:0071695)	7.2	5.46E-07
lipid localization (GO:0010876)	7.13	2.37E-02
developmental maturation (GO:0021700)	6.75	1.45E-06
seed development (GO:0048316)	5.48	1.23E-20
fruit development (GO:0010154)	5.27	6.90E-20
response to abscisic acid (GO:0009737)	4.59	5.25E-11
response to alcohol (GO:0097305)	4.57	1.82E-12
response to cold (GO:0009409)	4.5	2.82E-03
response to water deprivation (GO:0009414)	4.39	5.23E-09
response to water (GO:0009415)	4.34	1.40E-09
**Endosperm at 24h**		
response to heat (GO:0009408)	22.93	6.23E-04
response to temperature stimulus (GO:0009266)	9.24	2.12E-02
response to abiotic stimulus (GO:0009628)	3.89	3.96E-03
response to chemical (GO:0042221)	3.39	7.08E-03
response to stress (GO:0006950)	3.24	1.28E-02
response to stimulus (GO:0050896)	2.33	2.26E-02
**Embryo at 48h**		
Unclassified (UNCLASSIFIED)	2.21	0.00E+00
**Endosperm at 48h**		
sno(s)RNA 3'-end processing (GO:0031126)	12.91	2.20E-02
sno(s)RNA processing (GO:0043144)	10.88	1.31E-02
sno(s)RNA metabolic process (GO:0016074)	10.88	1.31E-02
small regulatory ncRNA 3'-end processing (GO:0043628)	9.39	3.12E-02
RNA phosphodiester bond hydrolysis (GO:0090501)	5.68	4.24E-02
RNA 3'-end processing (GO:0031123)	4.97	5.31E-03
mitochondrial RNA metabolic process (GO:0000959)	4.73	4.10E-02
seed maturation (GO:0010431)	4.64	5.28E-03
rRNA metabolic process (GO:0016072)	3.59	1.62E-07
rRNA processing (GO:0006364)	3.43	4.36E-06
ribosome biogenesis (GO:0042254)	3.07	1.61E-06
ribonucleoprotein complex biogenesis (GO:0022613)	3.02	7.72E-08
ncRNA processing (GO:0034470)	2.93	3.01E-07
RNA modification (GO:0009451)	2.83	1.26E-03
ncRNA metabolic process (GO:0034660)	2.74	2.78E-07
seed development (GO:0048316)	2.63	1.65E-18
response to heat (GO:0009408)	2.59	3.18E-02
fruit development (GO:0010154)	2.55	1.30E-17
RNA processing (GO:0006396)	2.26	1.23E-06
RNA metabolic process (GO:0016070)	2.06	1.54E-08

c

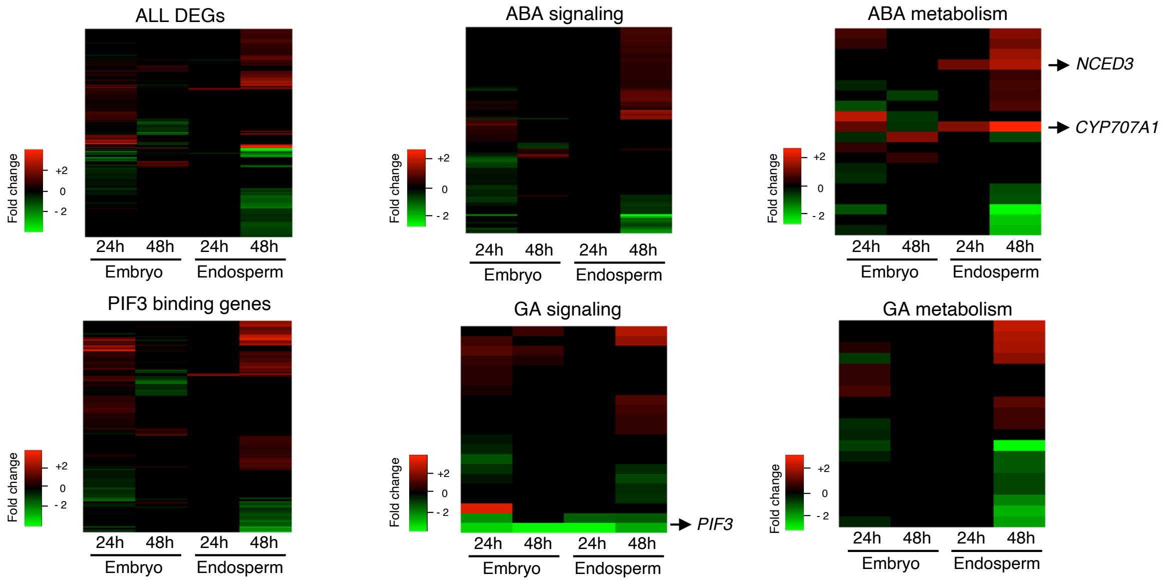

Supplementary Figure 4. PIF3 regulates largely different sets of genes in the endosperm and in the embryo.
(a) Venn diagrams shows the number of genes that are up-regulated or down-regulated at 24h and 48h after imbibition in *pif3* embryo and endosperm relative to WT embryo and endosperm, respectively. (b) GO enrichments of up-regulated (left) and down-regulated (right) genes at 24h and 48h after imbibition in *pif3* embryo and endosperm relative to WT embryo and endosperm, respectively. For the embryo at 24h and the endosperm at 48h, only top 20 GO terms for fold-enrichment are shown in the figure. Complete list of enriched GO terms is provided in Supplementary Data 1. Fisher's exact test (one-tailed) with the Bonferroni correction for multiple testing is used for the analysis.
(c) Heatmap representations of the log2 fold changes (*pif3*/WT) for all DEGs (differentially expressed genes in *pif3*), PIF3-binding genes, genes related to ABA and GA metabolism and signaling. Only the genes which were significantly ($p<0.05$) up-regulated or down-regulated in *pif3* relative to WT in at least one time point for a given tissue are shown. A complete gene list of PIF3-binding genes, genes related to ABA and GA metabolism and signaling including the genes that showed no significant expression changes in *pif3* is provided in Supplementary Data 1.

Supplementary Figure 5

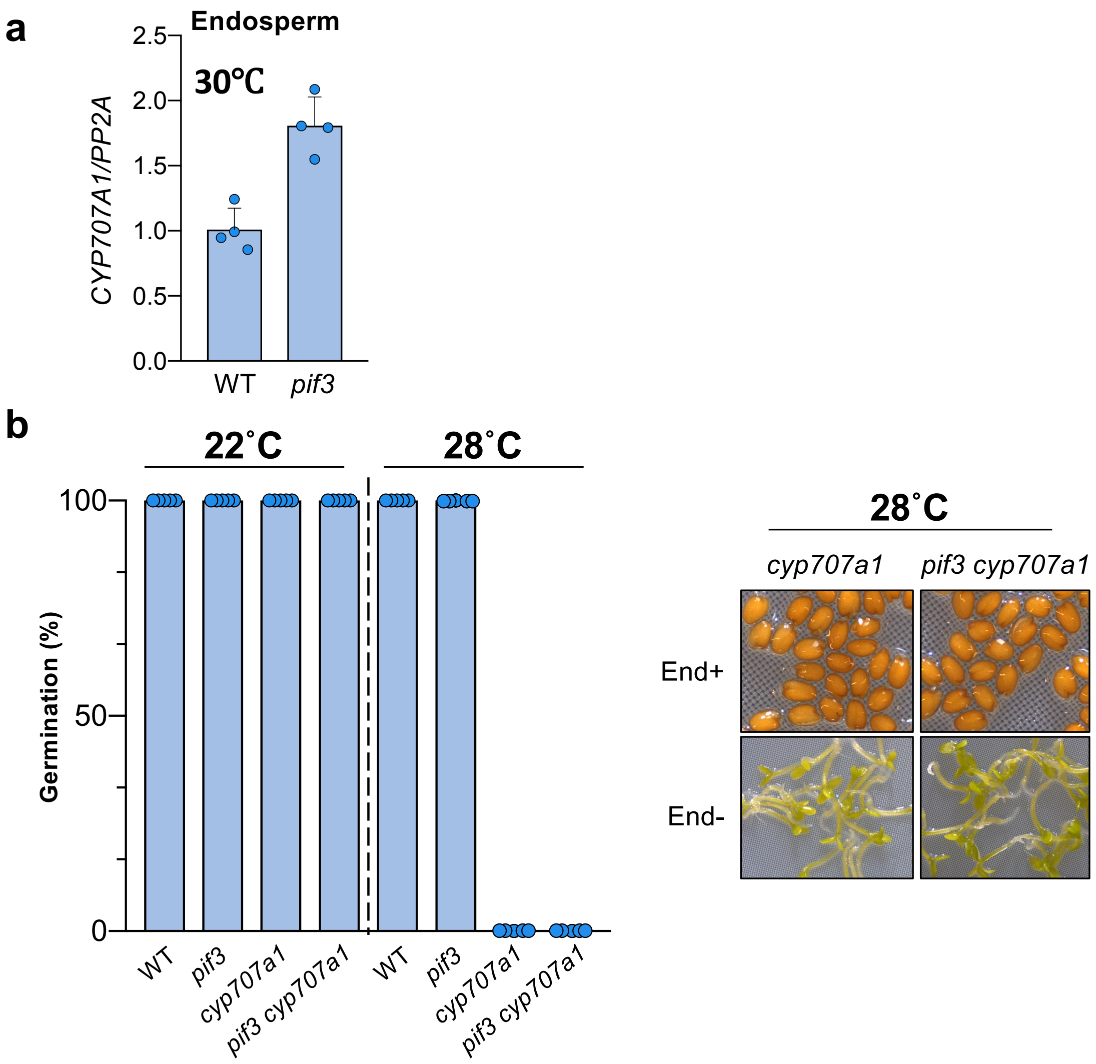

Supplementary Figure 5. PIF3 regulates largely different sets of genes in the endosperm and in the embryo.

a. Relative CYP707A1 mRNA accumulation in WT and pif3 endosperms dissected from seeds after 48h upon seed imbibition under white light (50 µmol/m2/s) at 30˚C. Total RNA was isolated from 150 endosperms. Histograms show the average expression value from 4 technical replicates. Data represent mean standard deviation (+). Expression levels were normalized to those of PP2A.

b. Average germination percentage of WT, *pif3*, *cyp707a1* (*cypa1*) and *pif3 cyp707a1* seeds cultured under white light (50 µmol/m2/s) for 3 days at 22˚C and 28˚C. 5 seed batches were analyzed. Pictures show *cyp707a1* and *pif3 cyp707a1* seeds (End+) and embryos dissected 4 hours after seed imbibition (End-) incubated for 3 days at 28˚C.

Supplementary Figure 6

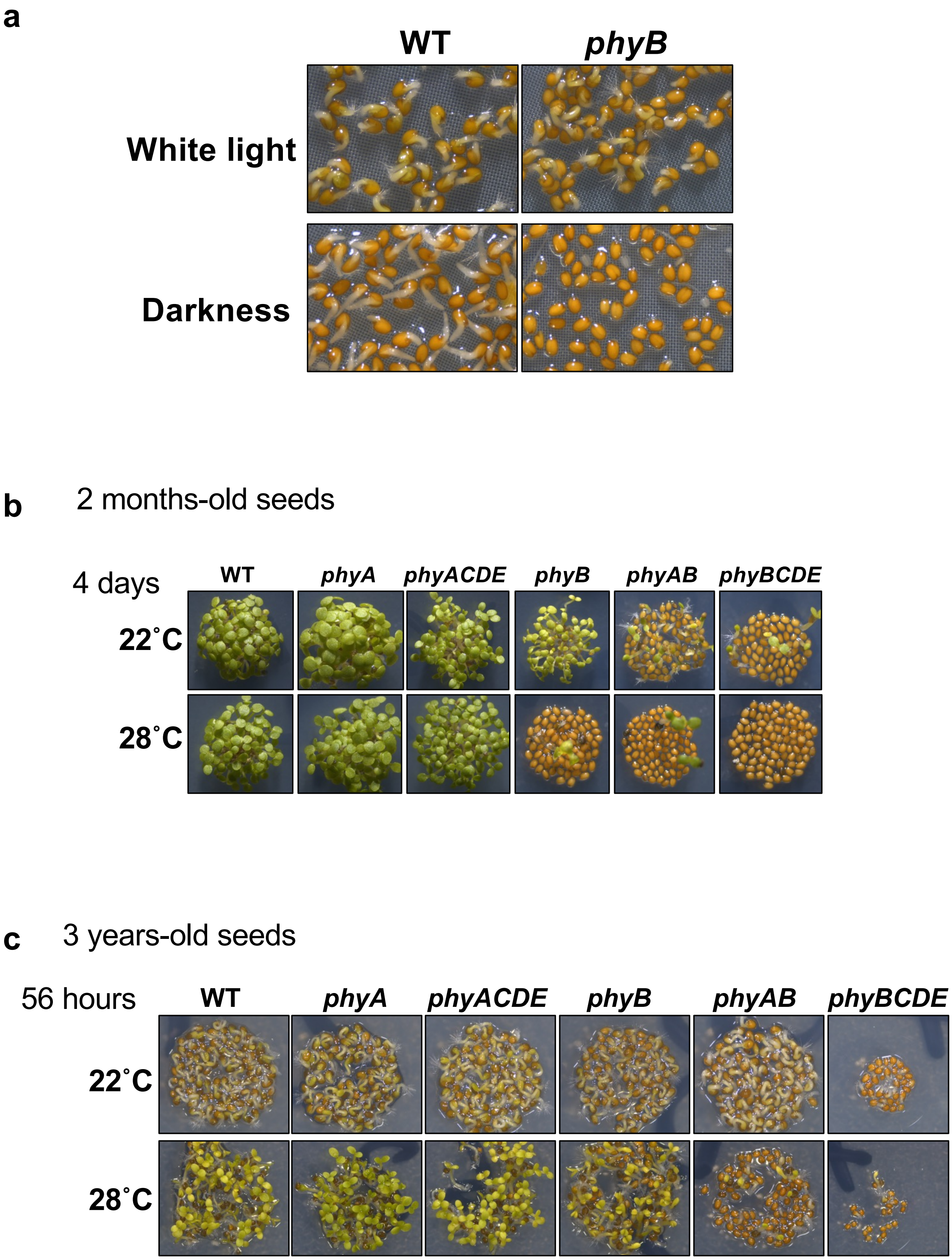

Supplementary Figure 6. Phytochrome-mediated thermoinhibition decreases in aging seeds.
a. Representative pictures of WT and *phyB* seeds cultured for 2 days at 22˚C in presence or absence of white light (50 µmol/m^2/s).
b. Representative pictures of 2-month-old WT, *phyA, phyB, phyAB*, *phyACDE* and *phyBCDE* seeds cultured for 4 days under white light (50 µmol/m^2/s) at 22˚C and 28˚C.
c. Same as b. with 3 years-old seeds.

Supplementary Figure 7

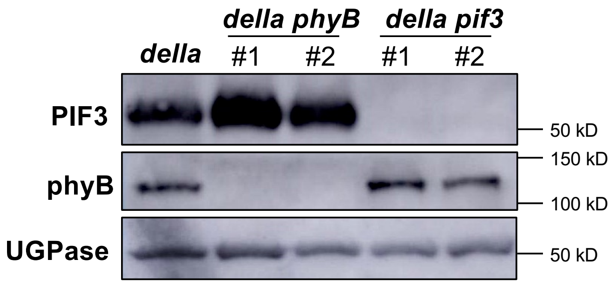

Supplementary Figure 7. Analysis of *phyB della* and *pif3 della* mutants generated by CRISPR/Cas9.

Protein gel blot analysis of PIF3 and PHYB levels in *della*, *della phyB* and *della pif3* seedlings cultivated in darkness for 2 days at 22˚C (#1 and #2 are repetitions). UGPase protein levels were used as a loading control.

Supplementary Figure 8

For Figure 1e

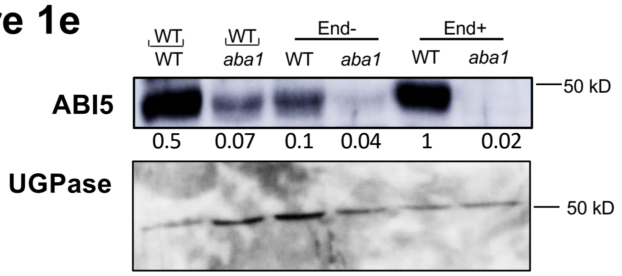

For Figure 2d

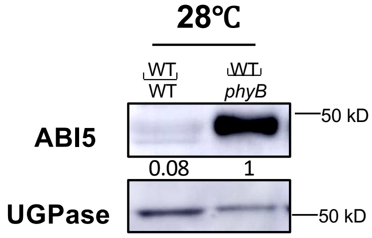

For Figure 2e

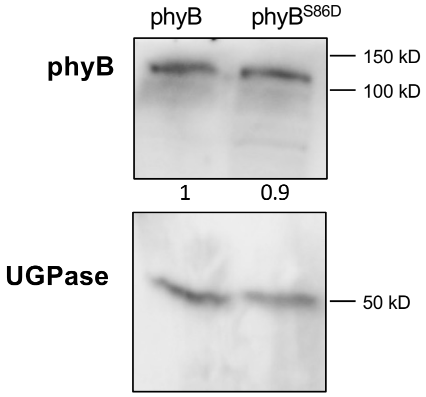

For Figure 2f

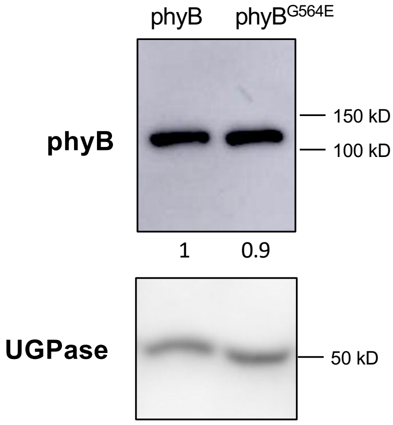

For Figure 3a

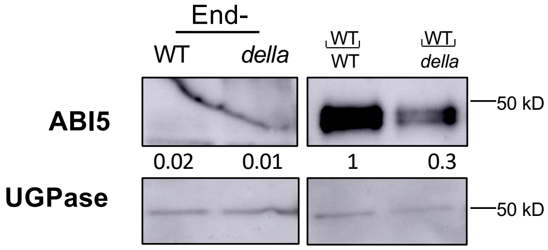

For Figure 3b

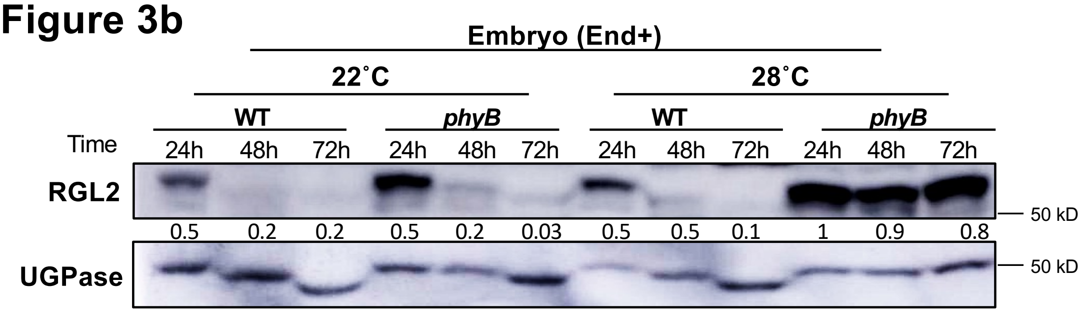

Supplementary Figure 8. Biological repetitions of protein blots presented in the main Figures.

Supplementary Figure 8

For Figure 3b

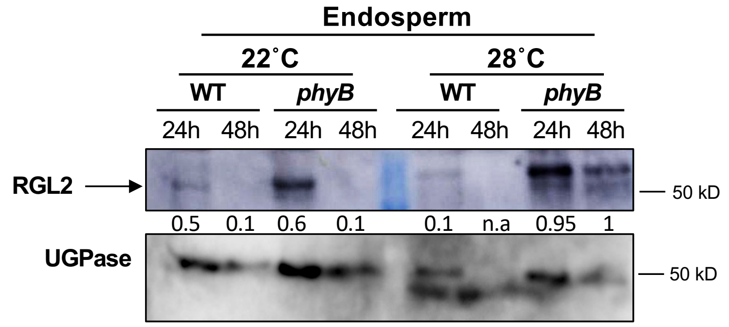

For Figure 3b (early time-points)

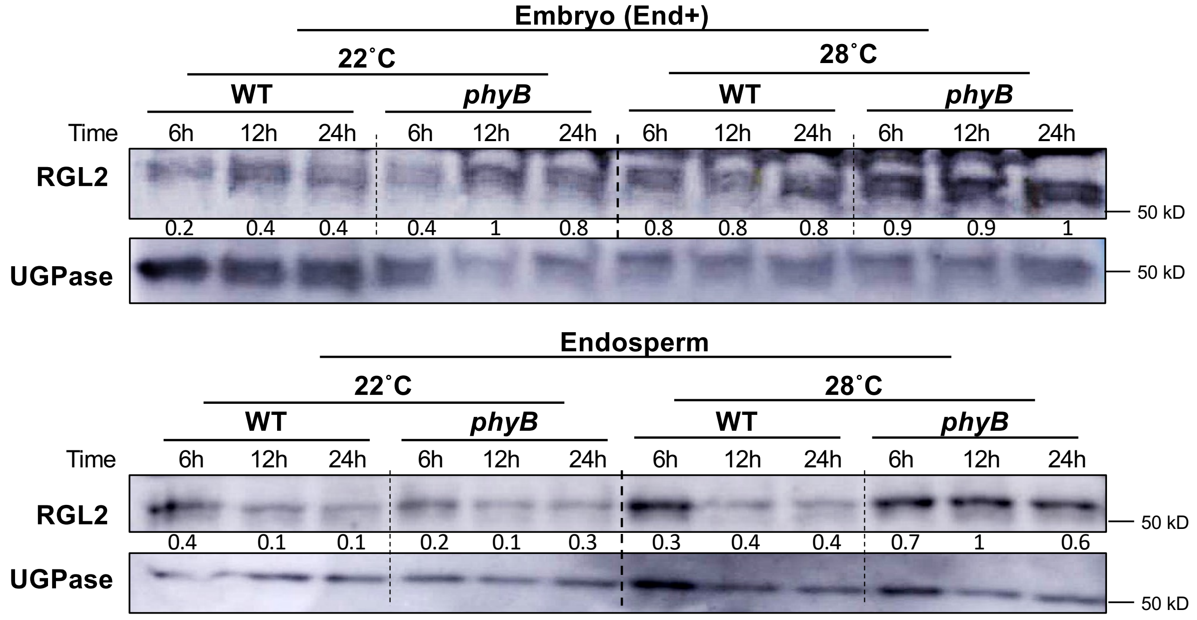

For Figure 3c

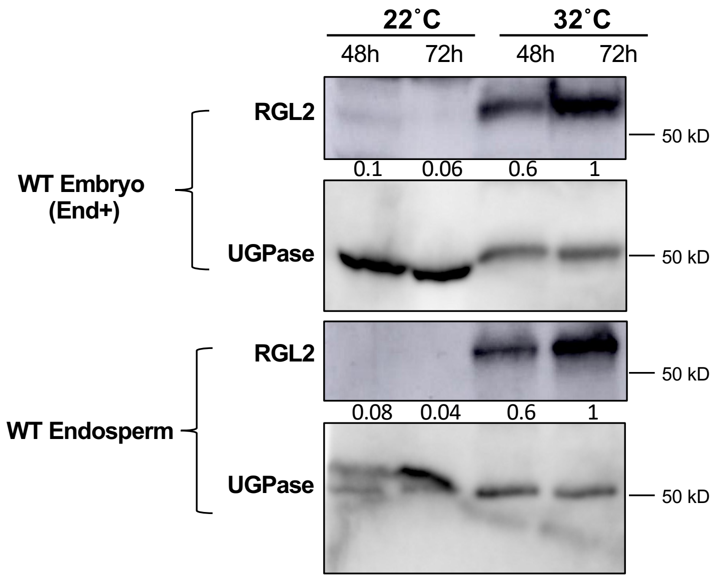

Supplementary Figure 8. Biological repetitions of protein blots presented in the main Figures.

Supplementary Figure 8

For Figure 3c (early time-points)

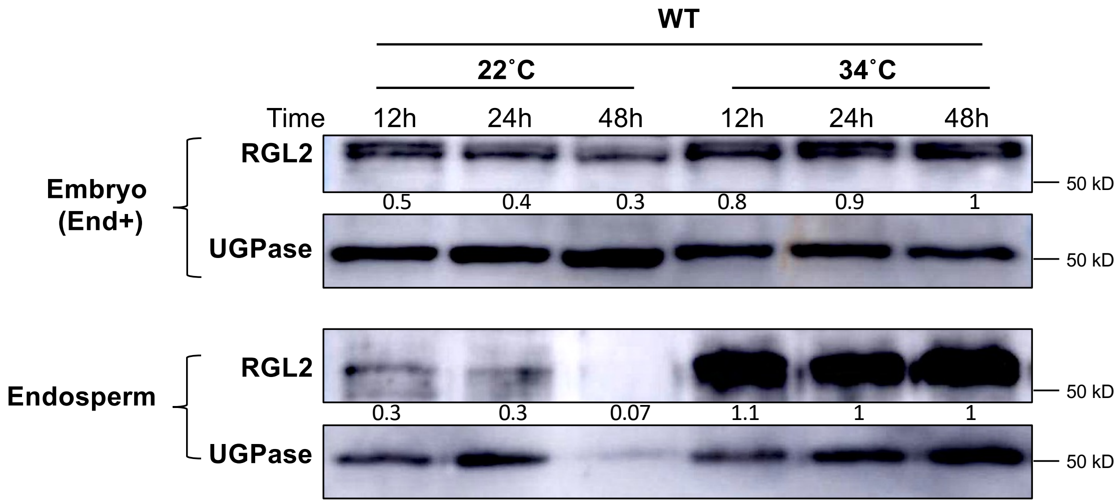

For Figure 3f

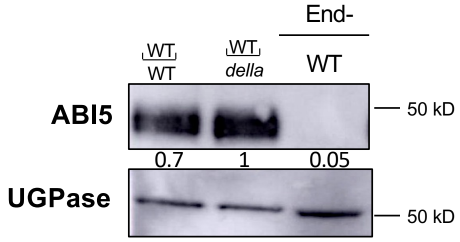

For Figure 5a

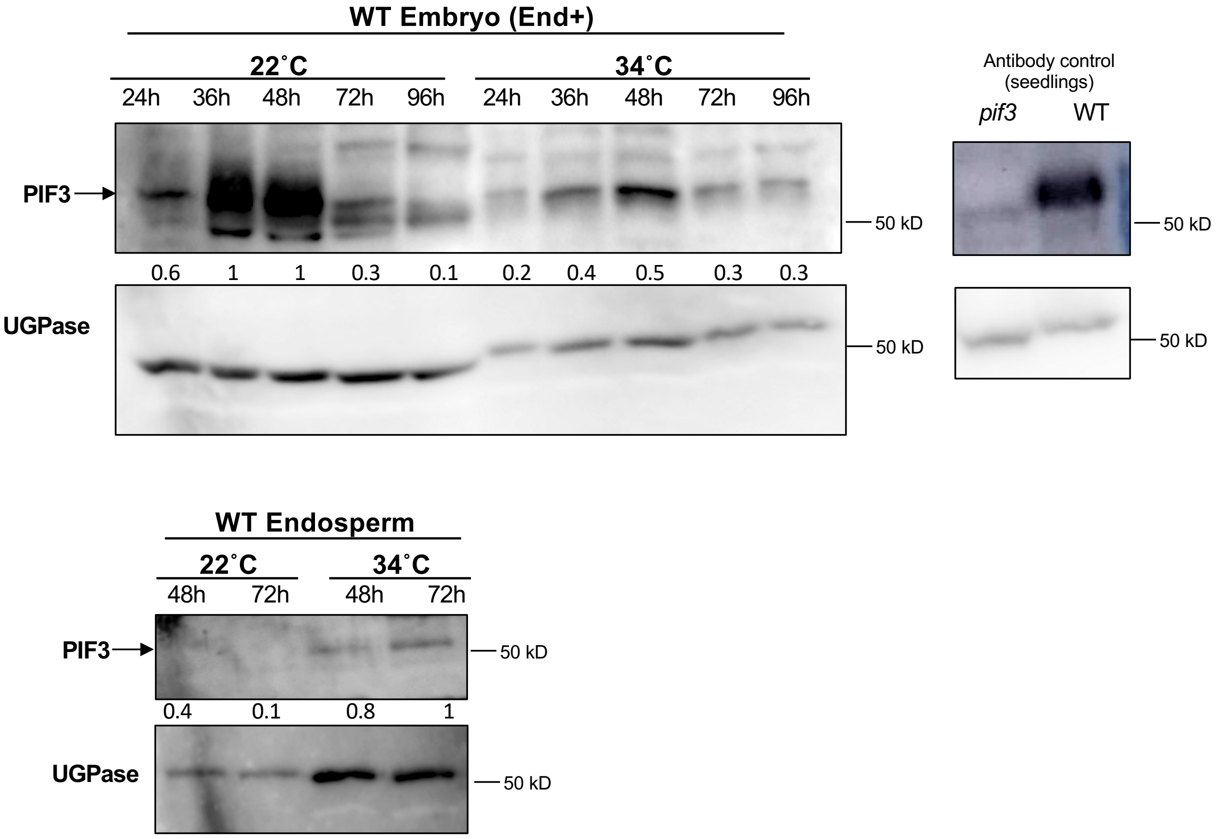

Supplementary Figure 8. Biological repetitions of protein blots presented in the main Figures.

Supplementary Figure 8

For Figure 5a (early time-points)

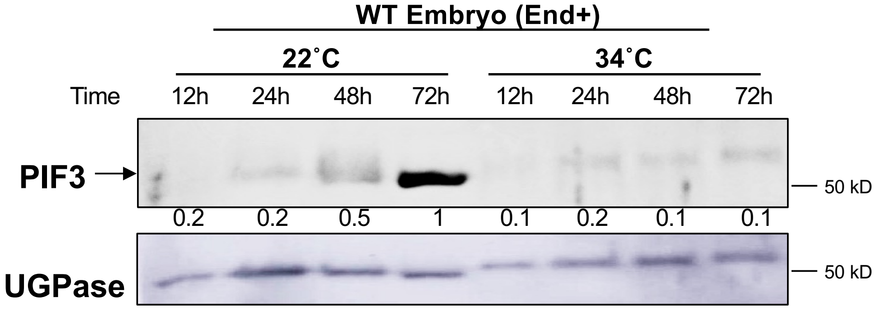

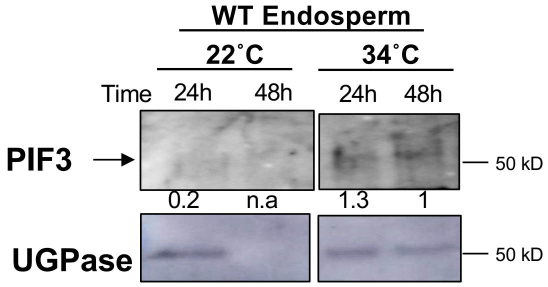

For Figure 5c

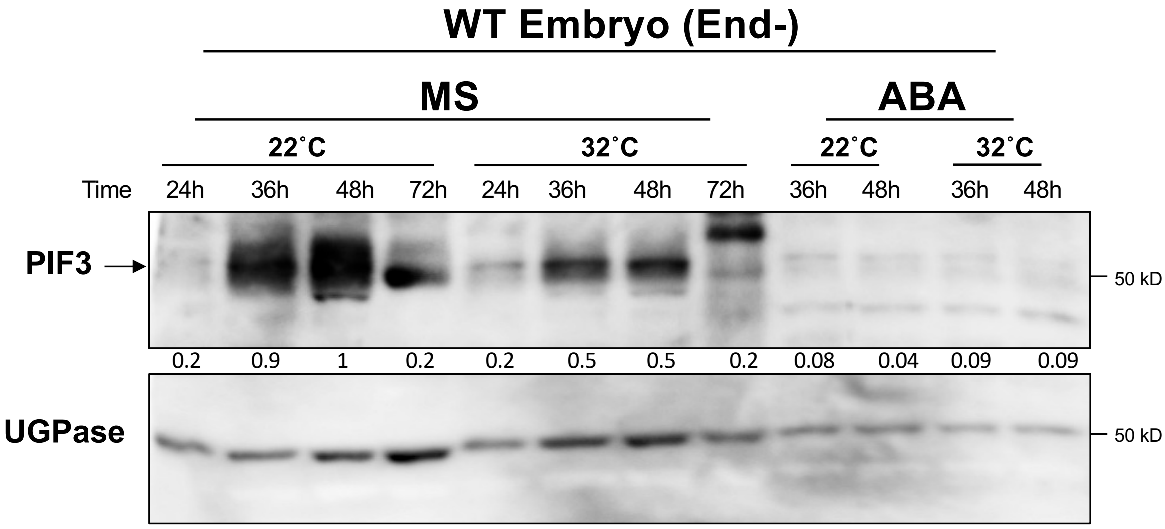

For Figure 5c (using 34˚C instead of 32˚C)

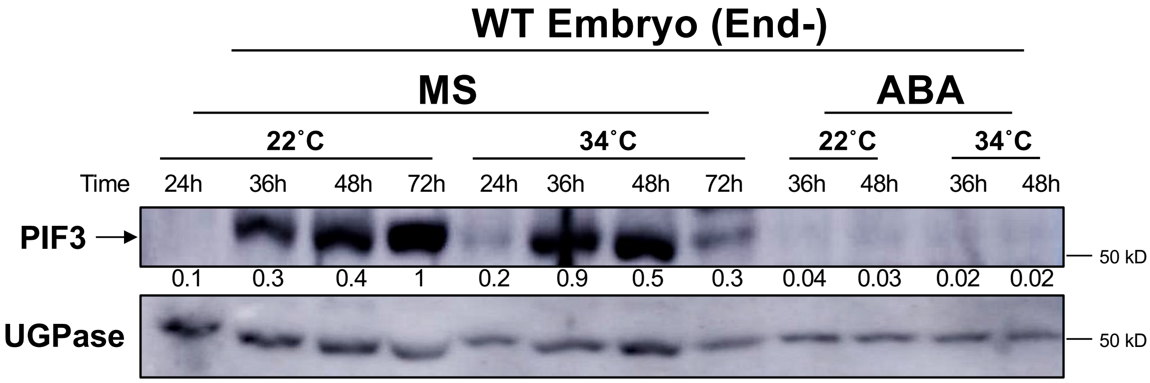

Supplementary Figure 8. Biological repetitions of protein blots presented in the main Figures.

Supplementary Figure 8

For Figure 5d

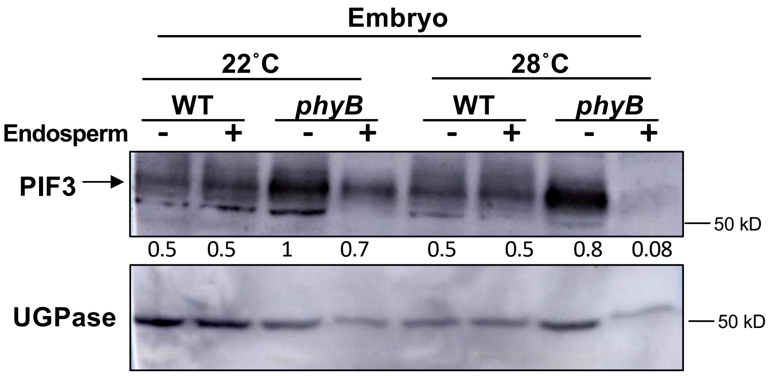

Supplementary Figure 8. Biological repetitions of protein blots presented in the main Figures.

Supplementary Figure 9

Figure 1e

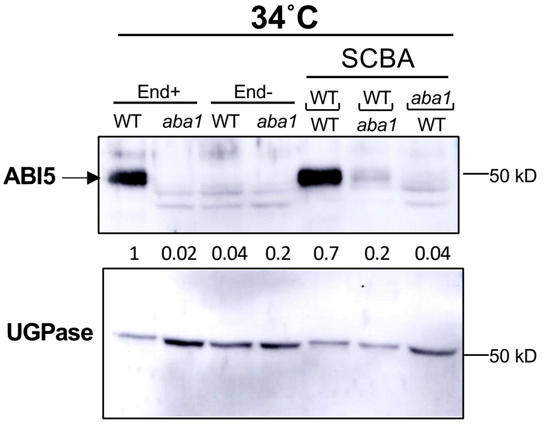

Repetition of Figure 1e (shown in suppl. Fig. 8)

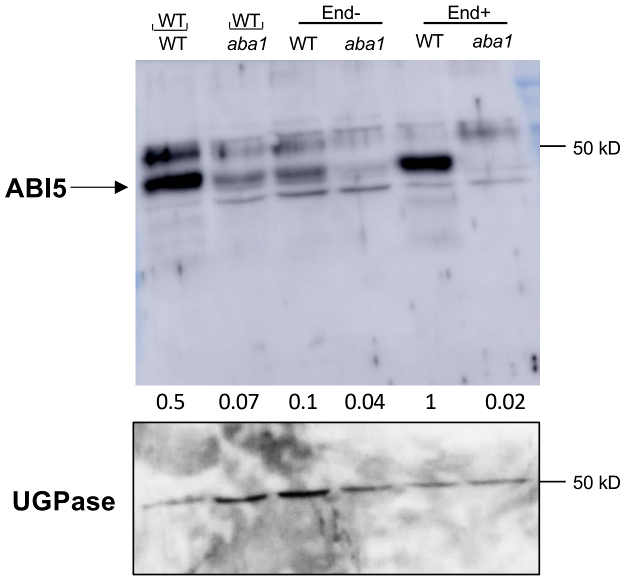

Supplementary Figure 9. Full, non-cropped protein blots presented in the main Figures and in the repetitions shown in Supplementary Figure 8.

Supplementary Figure 9

Figure 2d

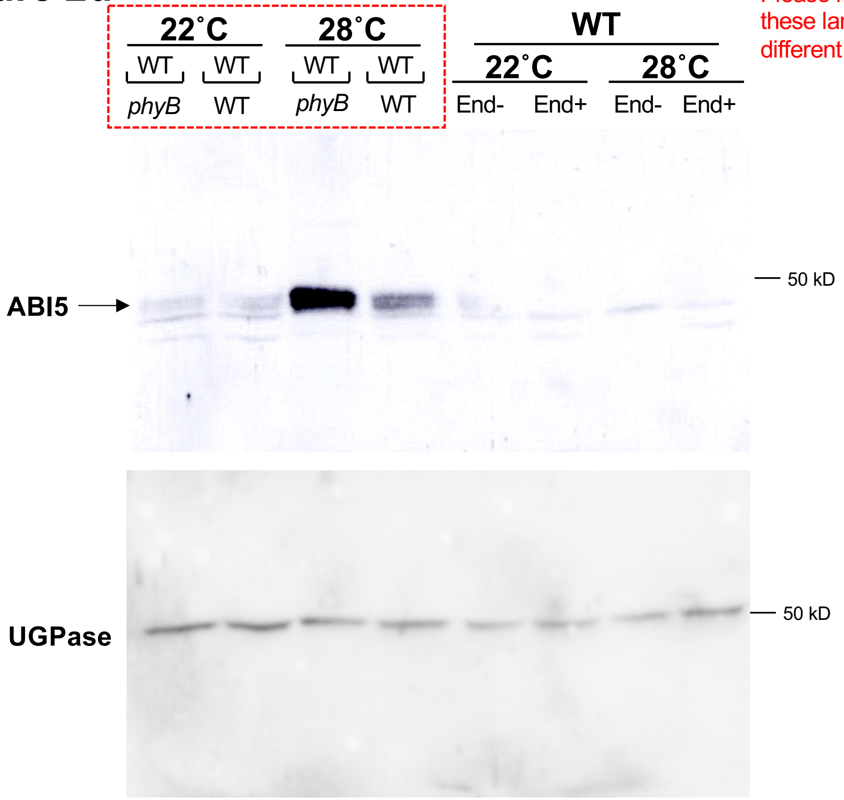

Please note that in the main figure these lanes are presented in a different order

Repetition of Figure 2d (shown in suppl. Fig. 8)

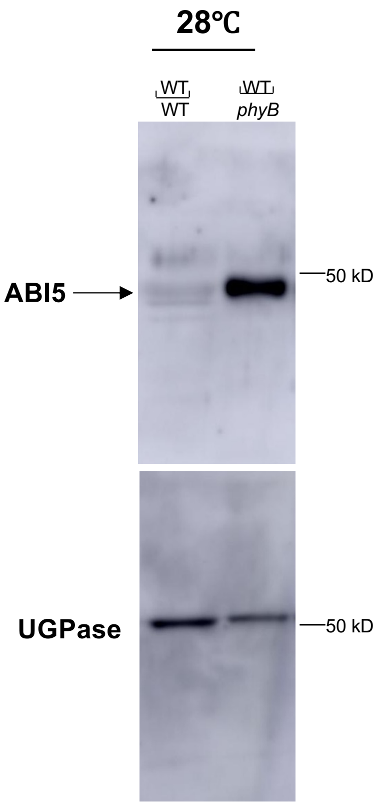

Supplementary Figure 9. Full, non-cropped protein blots presented in the main Figures and in the repetitions shown in Supplementary Figure 8.

Supplementary Figure 9

Figure 2e

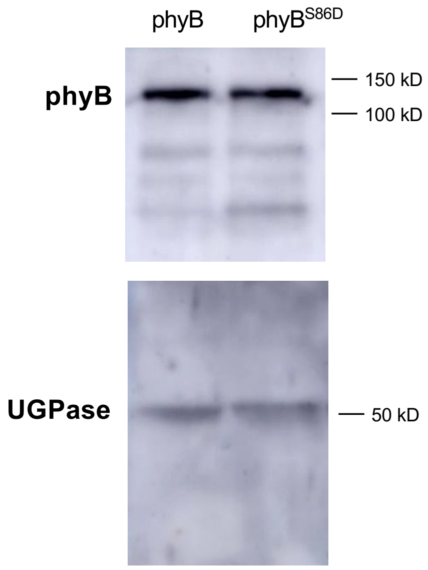

Figure 2f

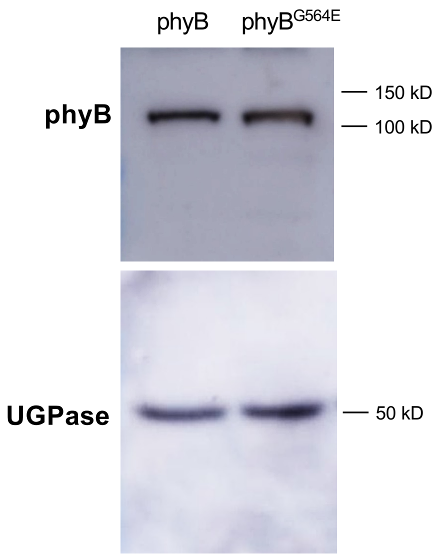

Repetition of Figure 2e (shown in suppl. Fig. 8)

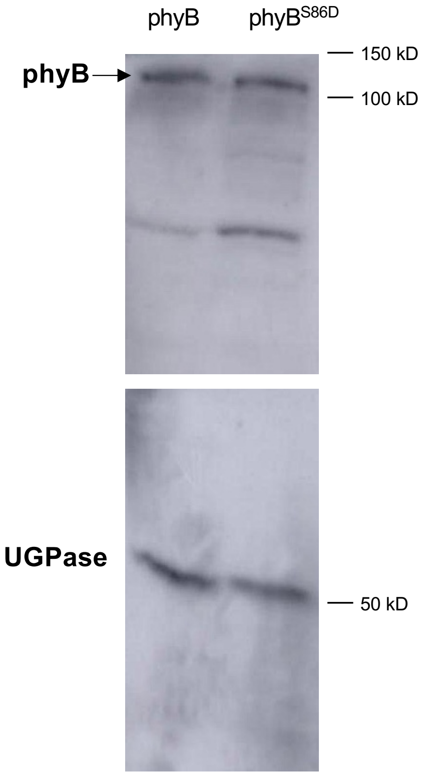

Repetition of Figure 2f (shown in suppl. Fig. 8)

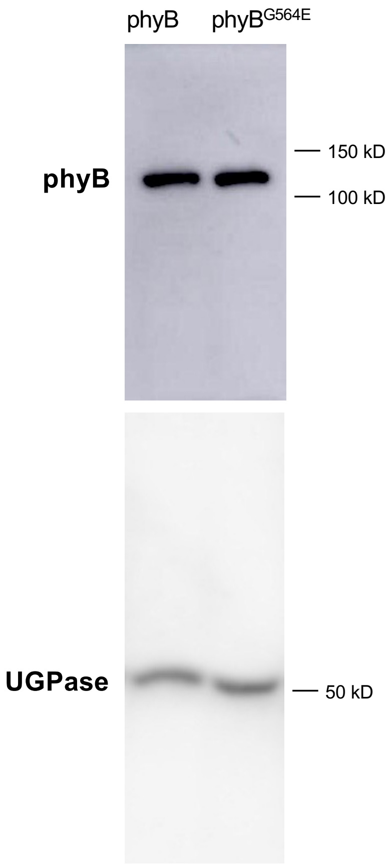

Supplementary Figure 9. Full, non-cropped protein blots presented in the main Figures and in the repetitions shown in Supplementary Figure 8.

Supplementary Figure 9

Figure 3a

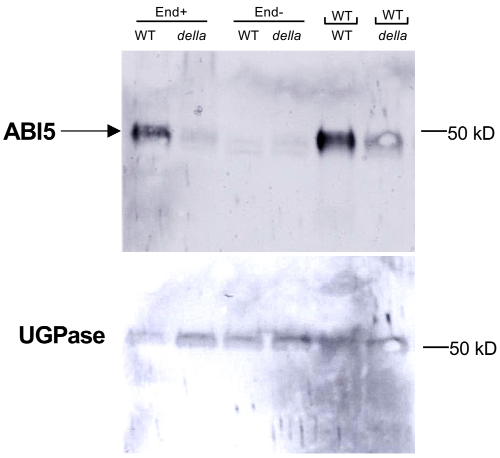

Repetition of Figure 3a (shown in suppl. Fig. 8)

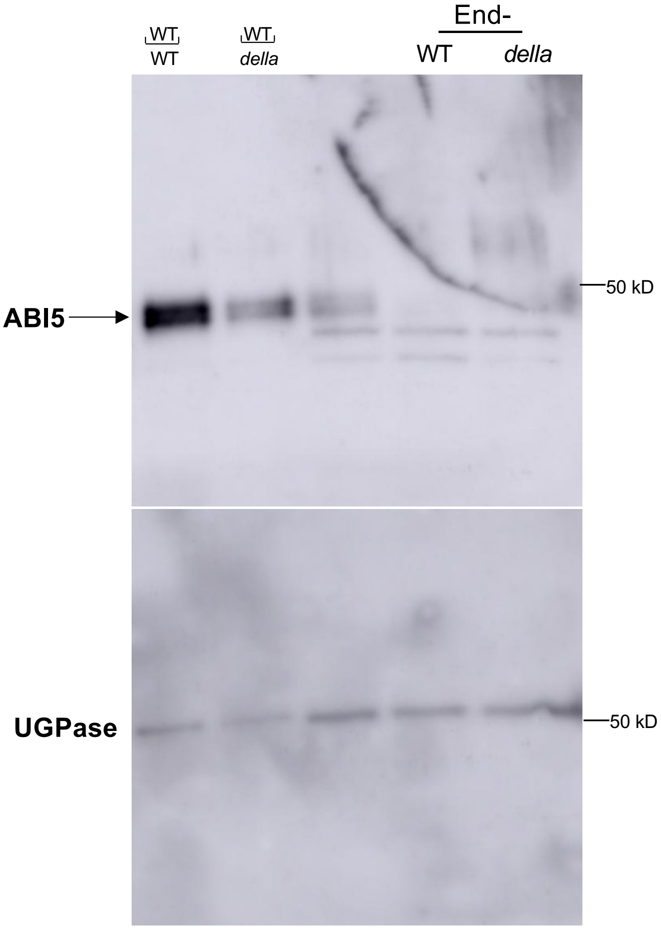

Supplementary Figure 9. Full, non-cropped protein blots presented in the main Figures and in the repetitions shown in Supplementary Figure 8.

Figure 3b

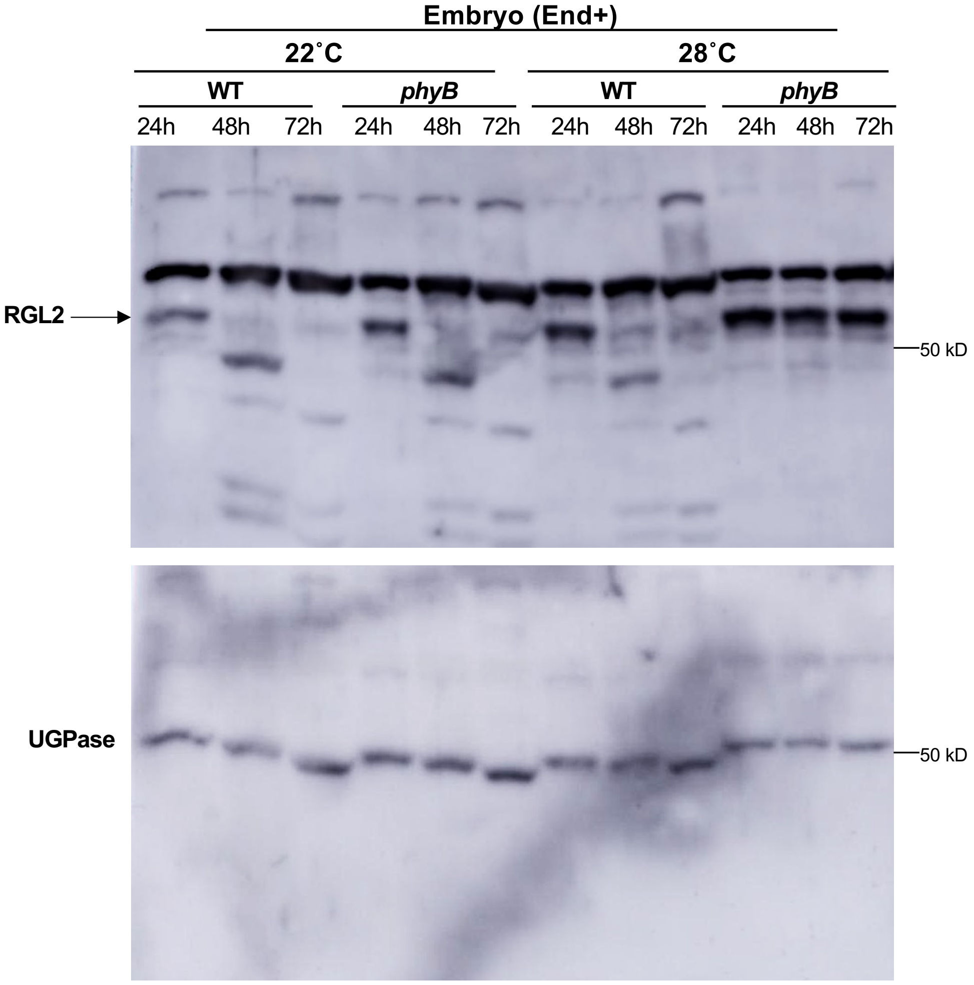

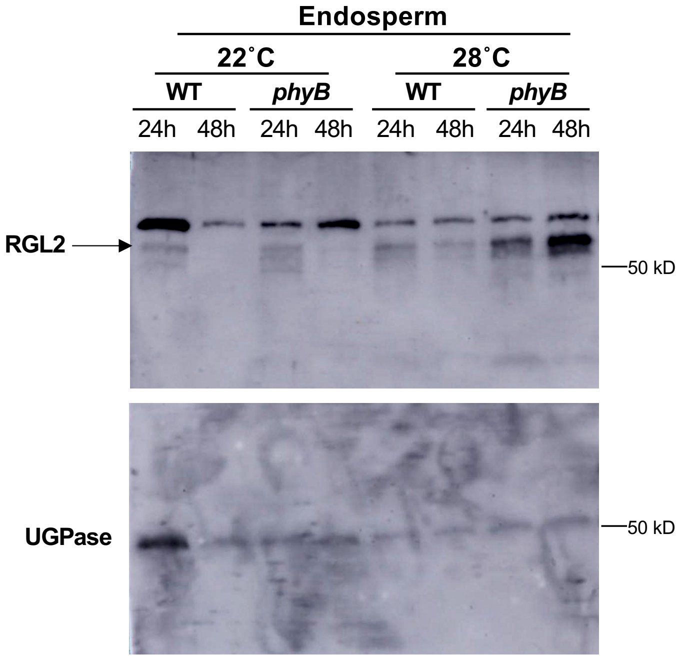

Supplementary Figure 9. Full, non-cropped protein blots presented in the main Figures and in the repetitions shown in Supplementary Figure 8.

Supplementary Figure 9

Repetition of Figure 3b (shown in suppl. Fig. 8)

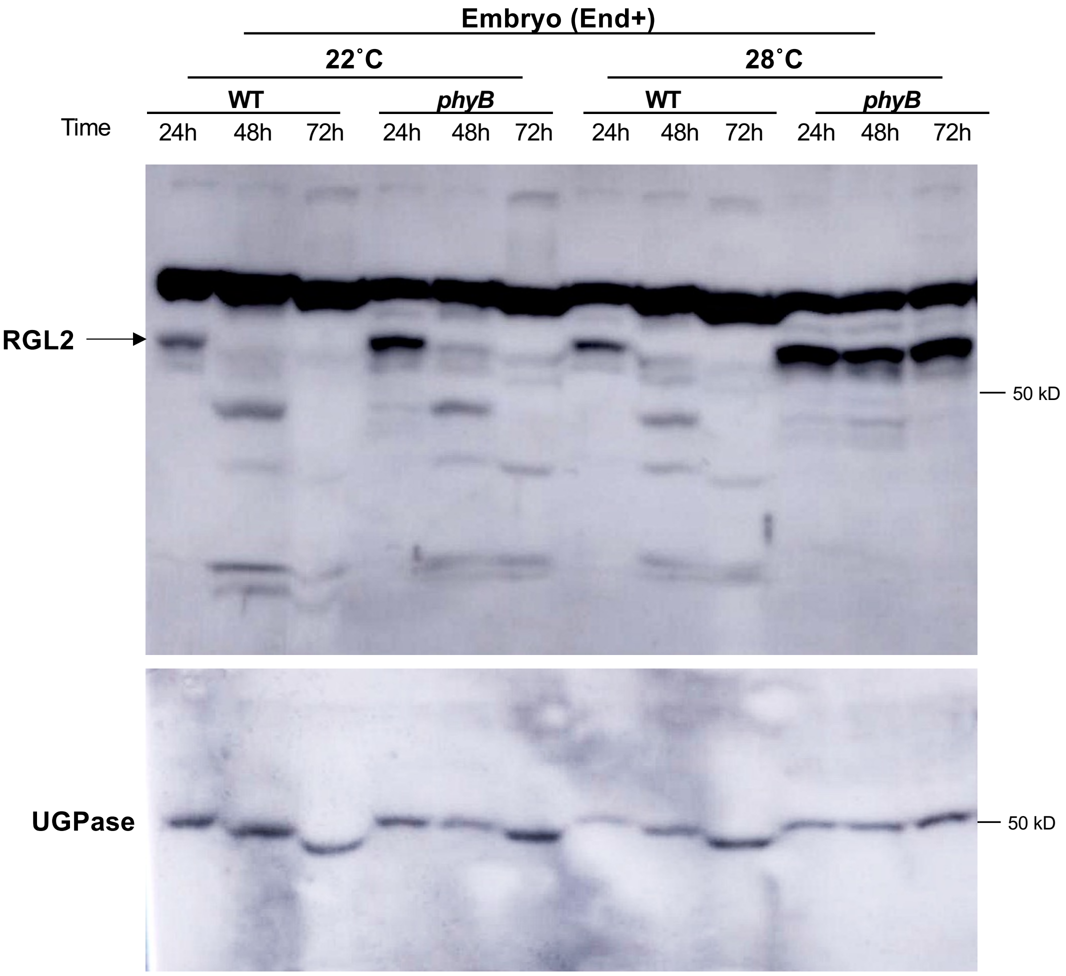

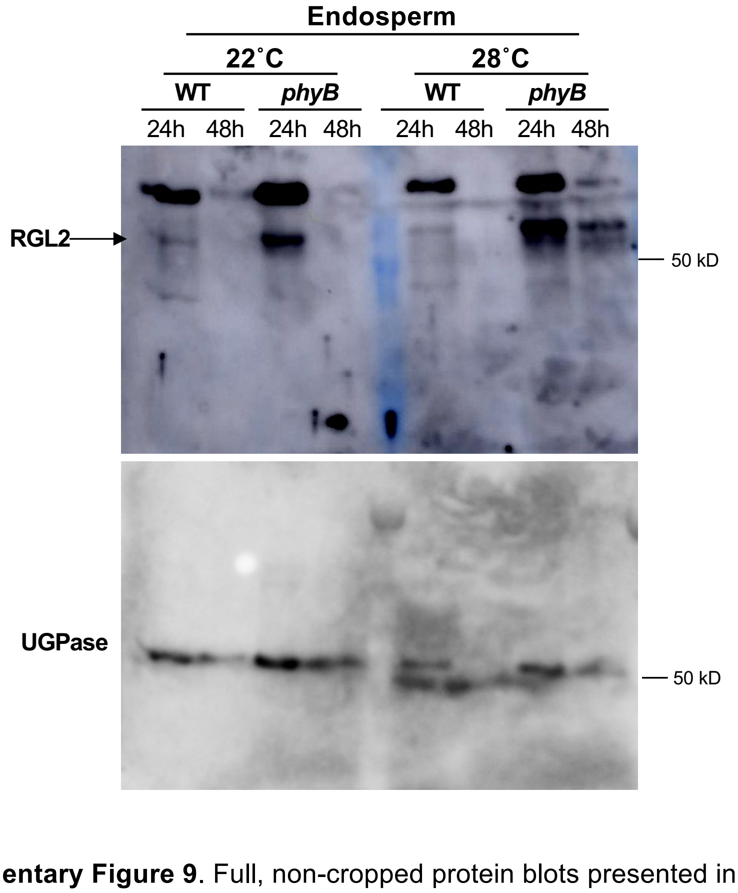

Supplementary Figure 9. Full, non-cropped protein blots presented in the main Figures and in the repetitions shown in Supplementary Figure 8.

Supplementary Figure 9

Repetition for Figure 3b (early time-points, shown in suppl. Fig. 8)

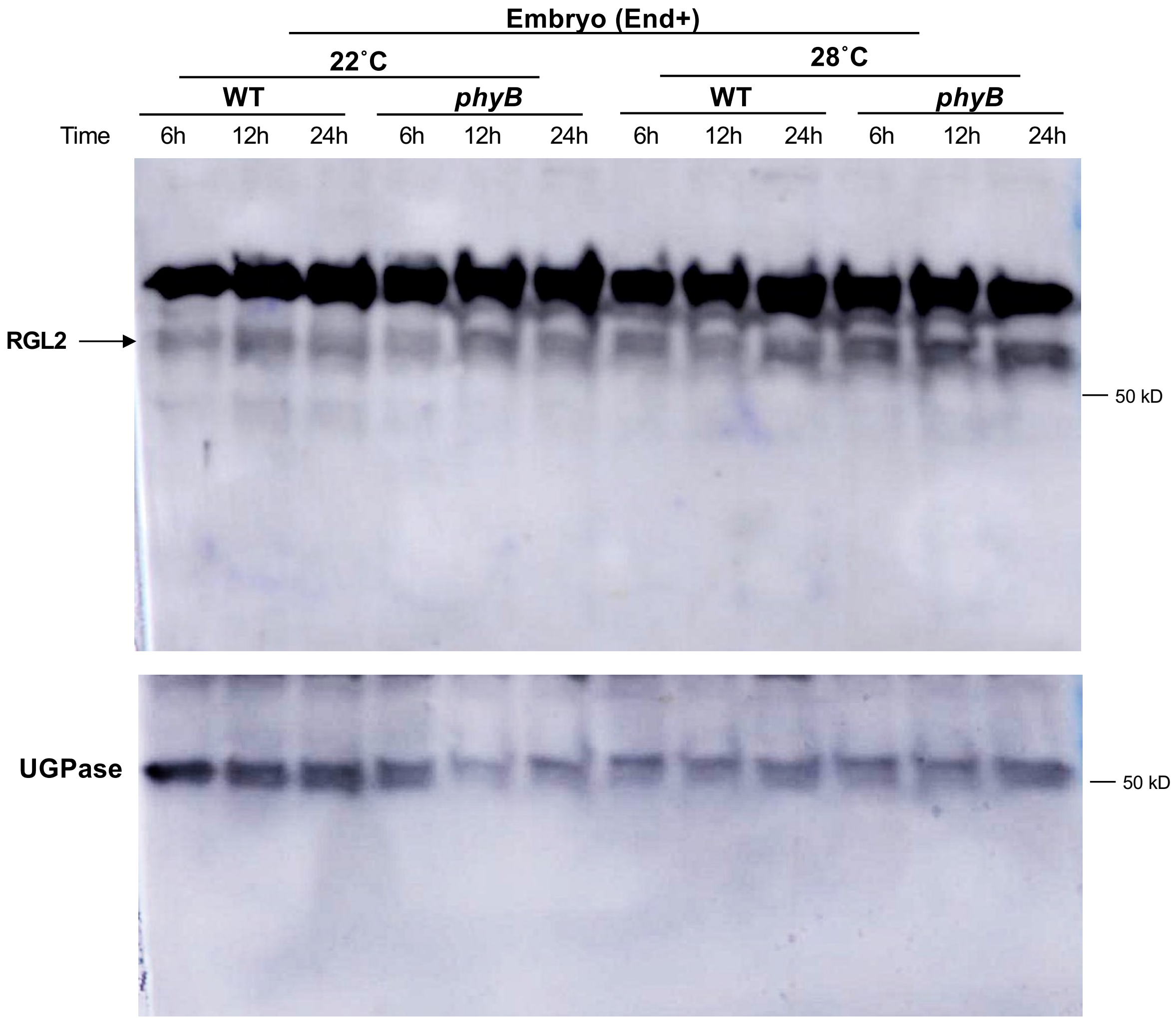

Supplementary Figure 9. Full, non-cropped protein blots presented in the main Figures and in the repetitions shown in Supplementary Figure 8.

Repetition for Figure 3b (early time-points, shown in suppl. Fig. 8)

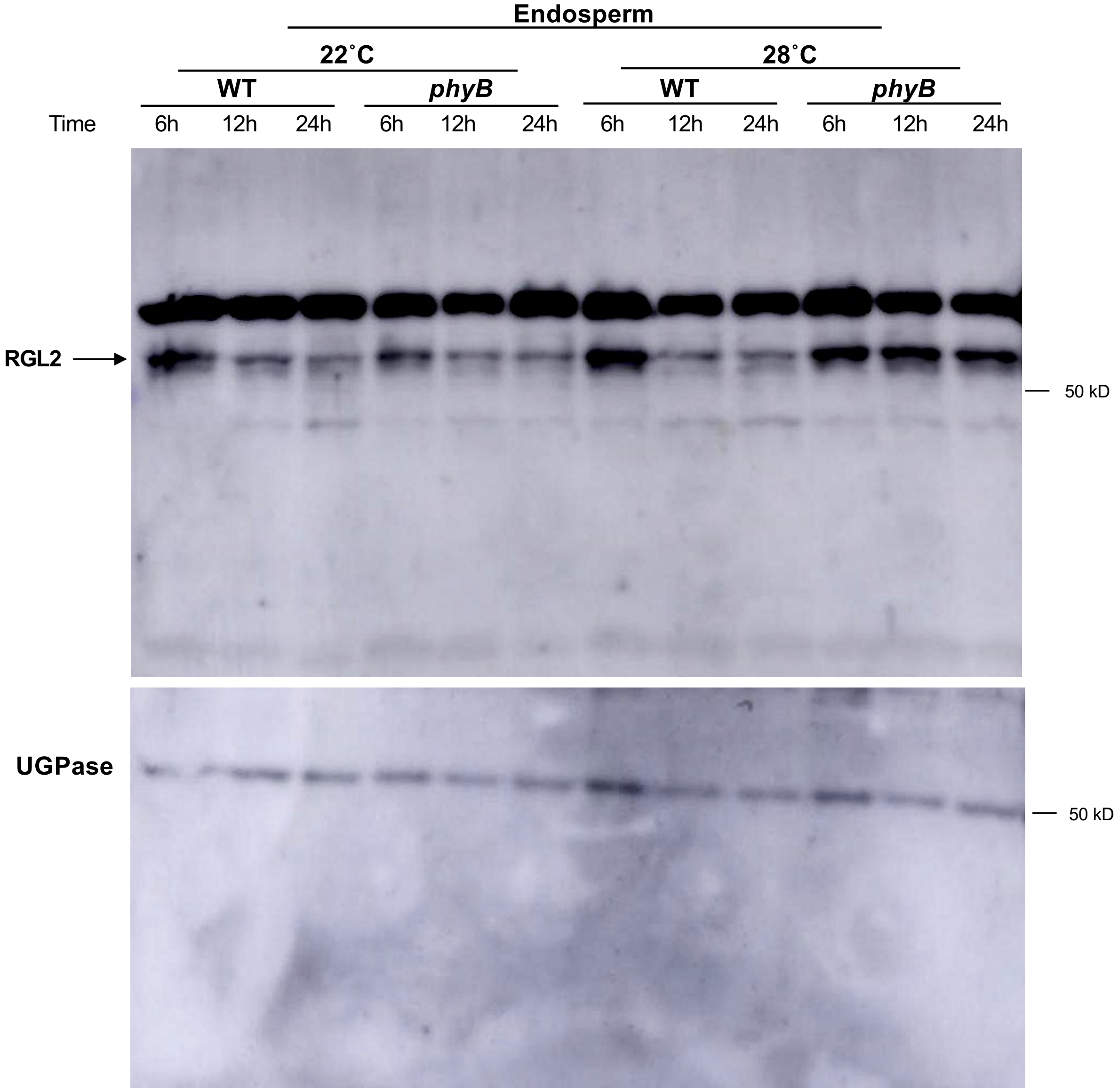

Supplementary Figure 9. Full, non-cropped protein blots presented in the main Figures and in the repetitions shown in Supplementary Figure 8.

Supplementary Figure 9

Figure 3c

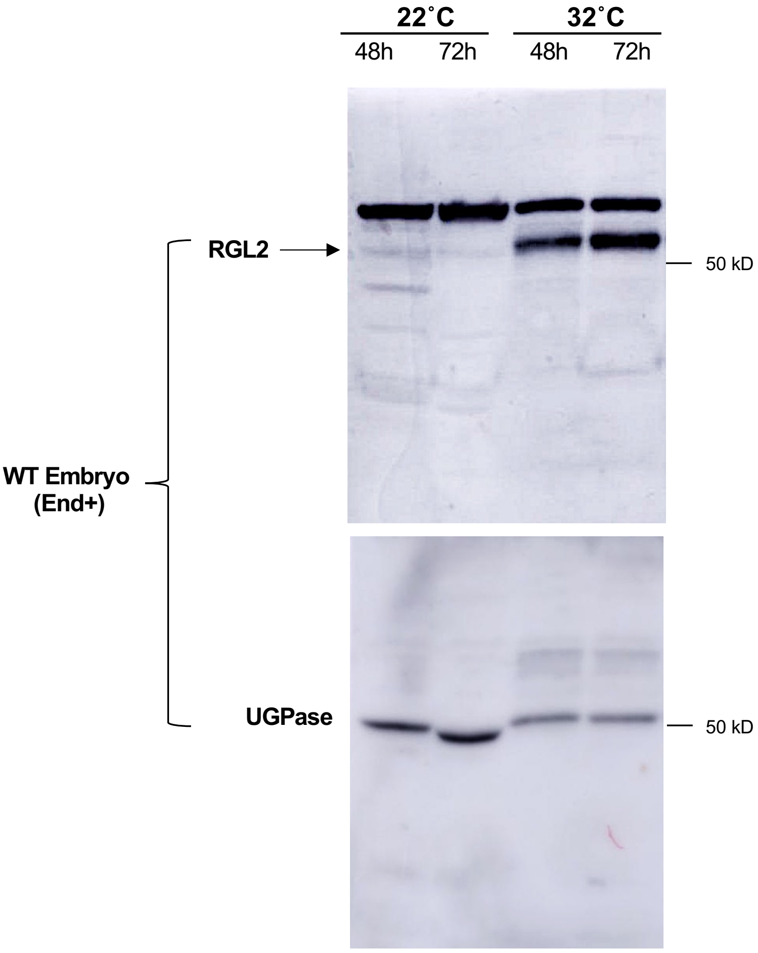

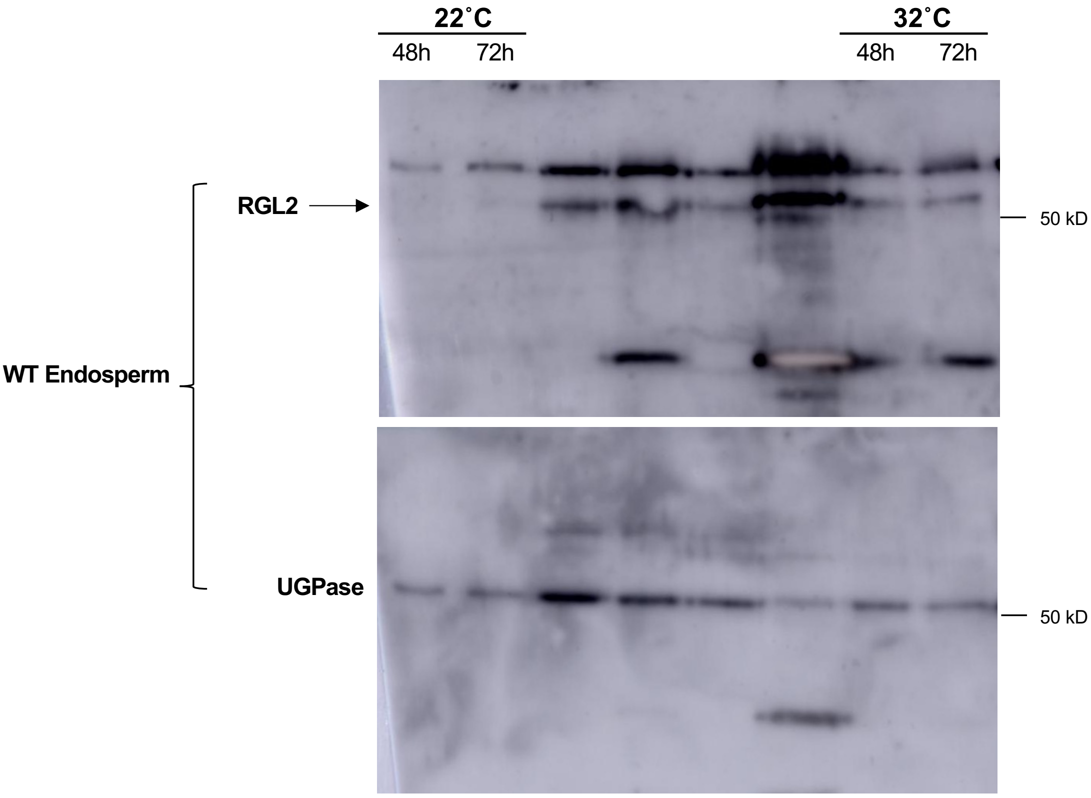

Supplementary Figure 9. Full, non-cropped protein blots presented in the main Figures and in the repetitions shown in Supplementary Figure 8.

Supplementary Figure 9

Repetition for Figure 3c (shown in suppl. Fig. 8)

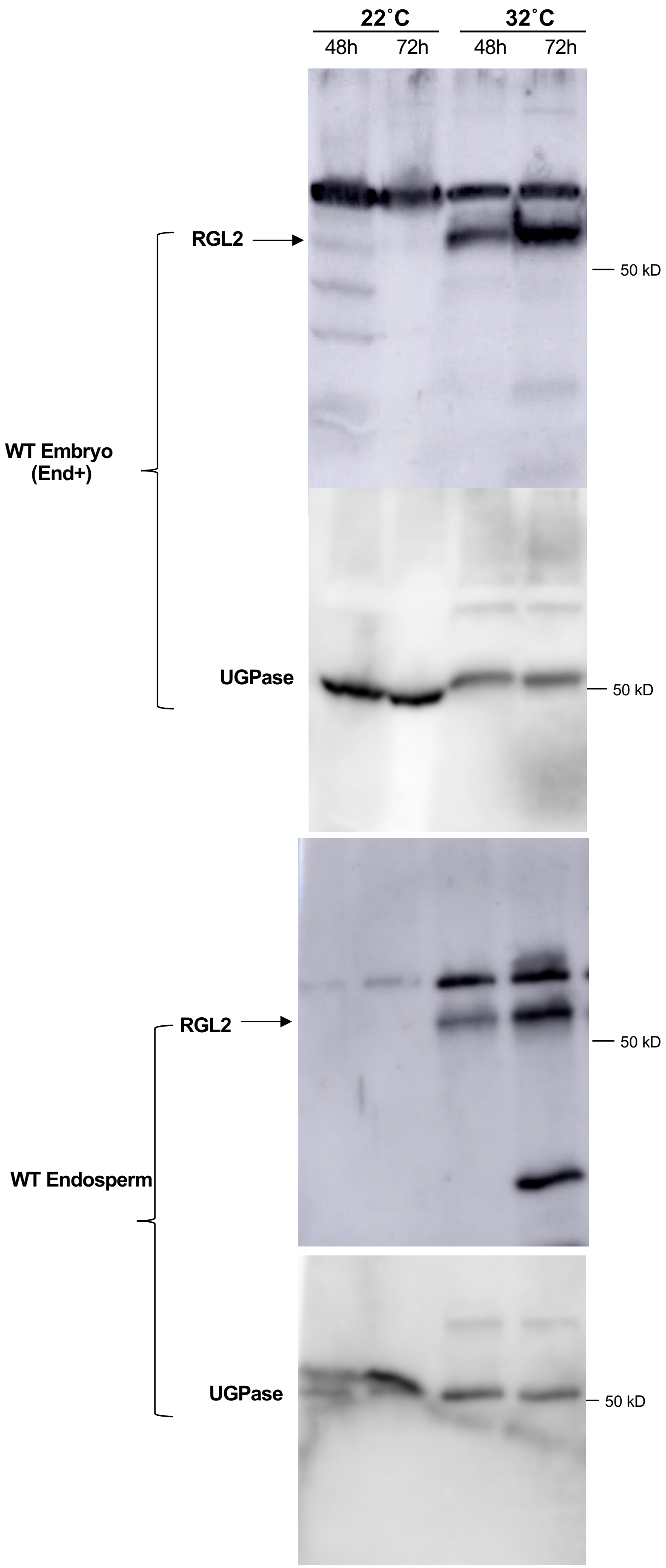

Supplementary Figure 9. Full, non-cropped protein blots presented in the main Figures and in the repetitions shown in Supplementary Figure 8.

Repetition for Figure 3c (early time-points, shown in suppl. Fig. 8)

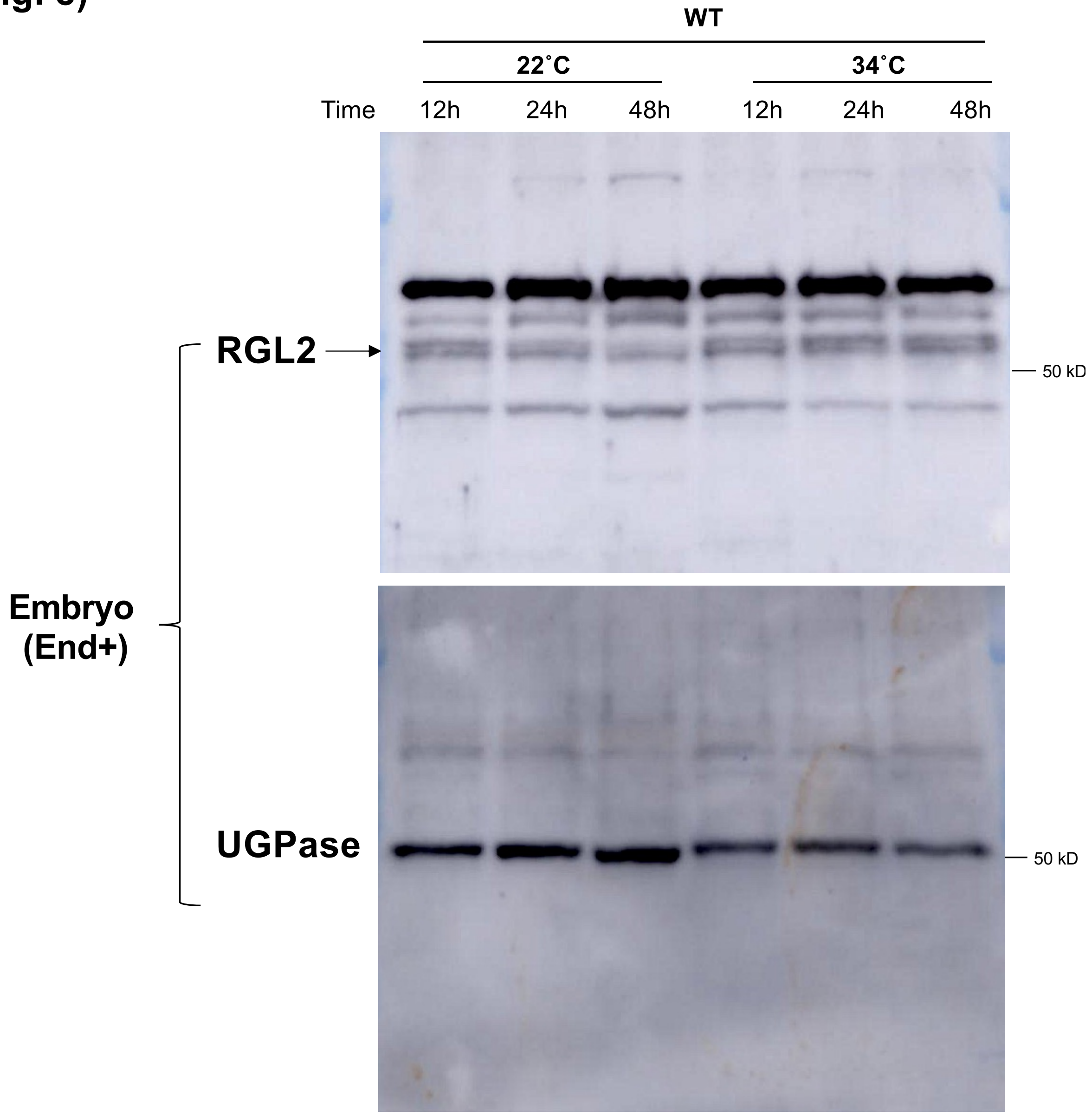

Supplementary Figure 9. Full, non-cropped protein blots presented in the main Figures and in the repetitions shown in Supplementary Figure 8.

Supplementary Figure 9

Repetition for Figure 3c (early time-points, shown in suppl. Fig. 8)

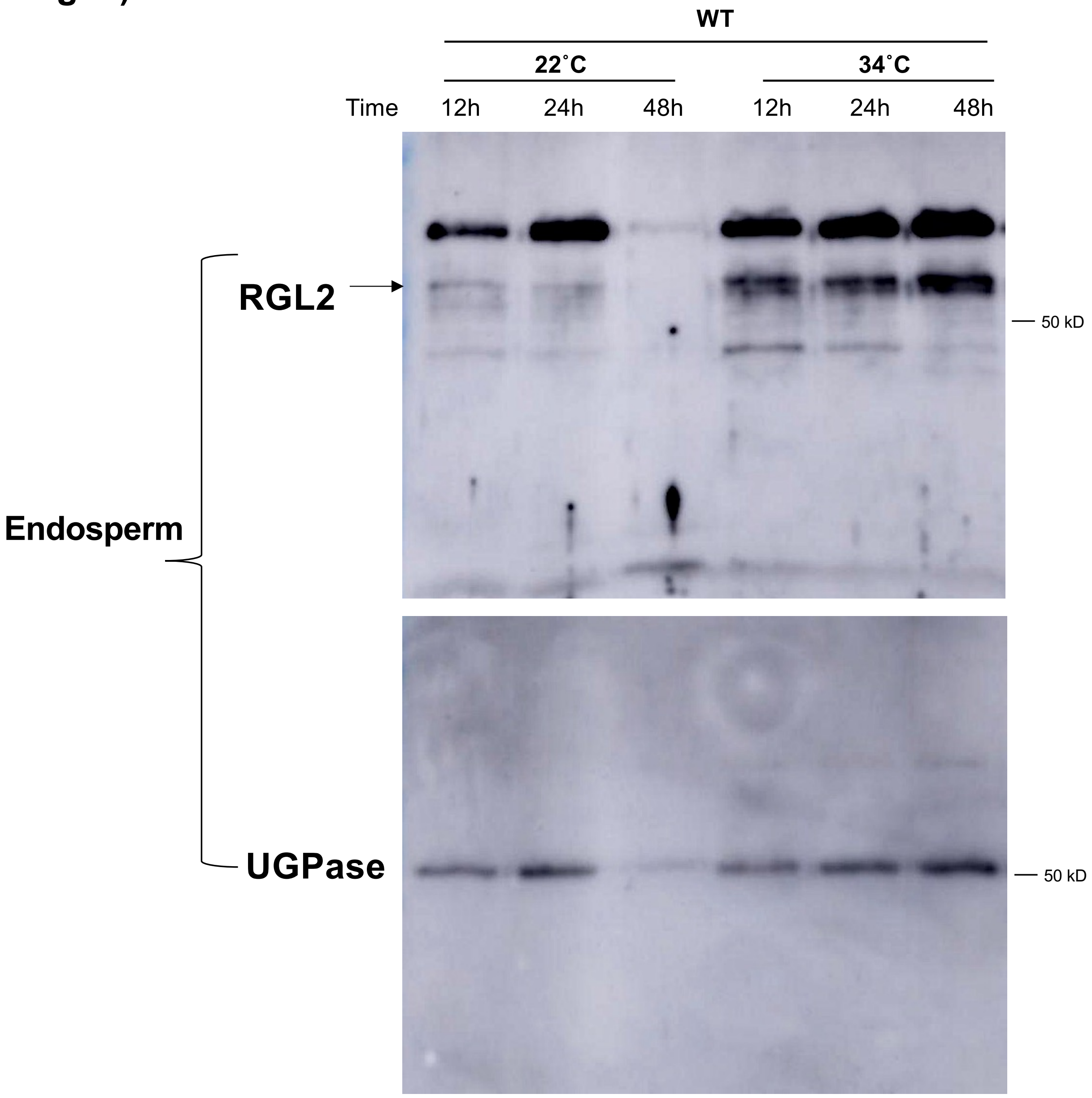

Supplementary Figure 9. Full, non-cropped protein blots presented in the main Figures and in the repetitions shown in Supplementary Figure 8.

Supplementary Figure 9

Figure 3f

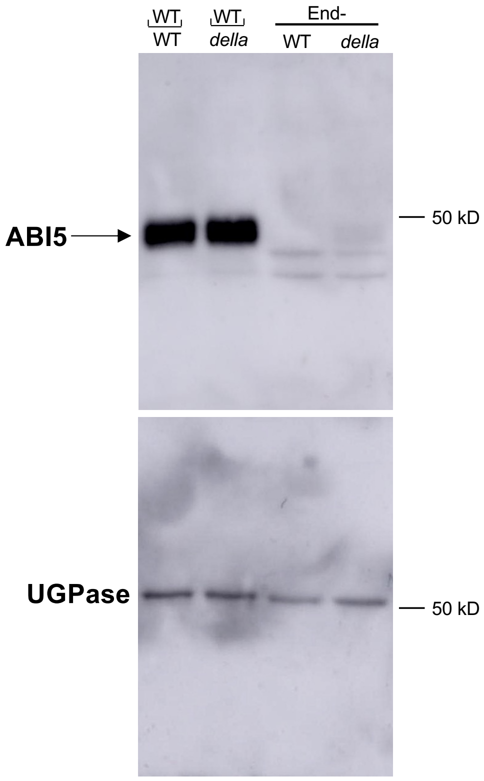

Repetition for Figure 3f (shown in suppl. Fig. 8)

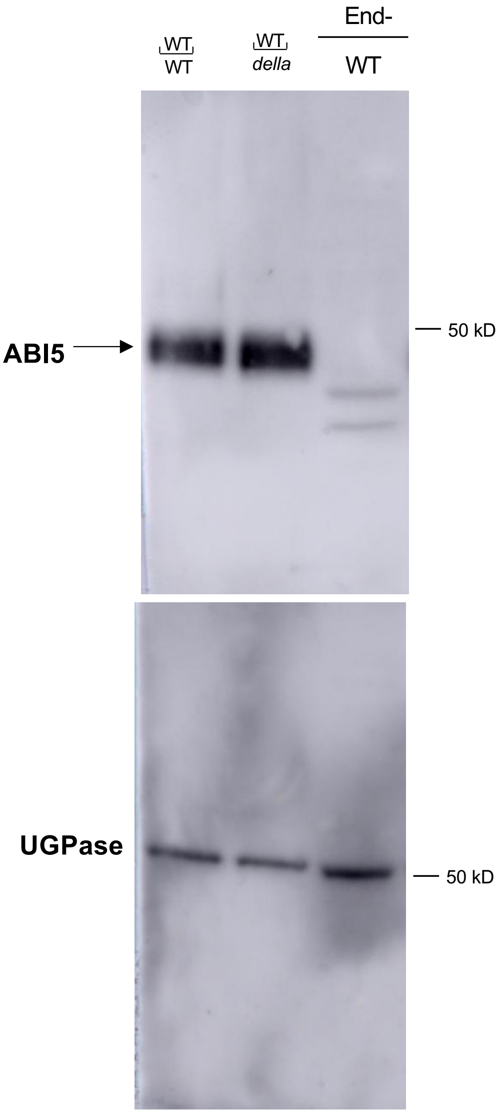

Supplementary Figure 9. Full, non-cropped protein blots presented in the main Figures and in the repetitions shown in Supplementary Figure 8.

Figure 5a

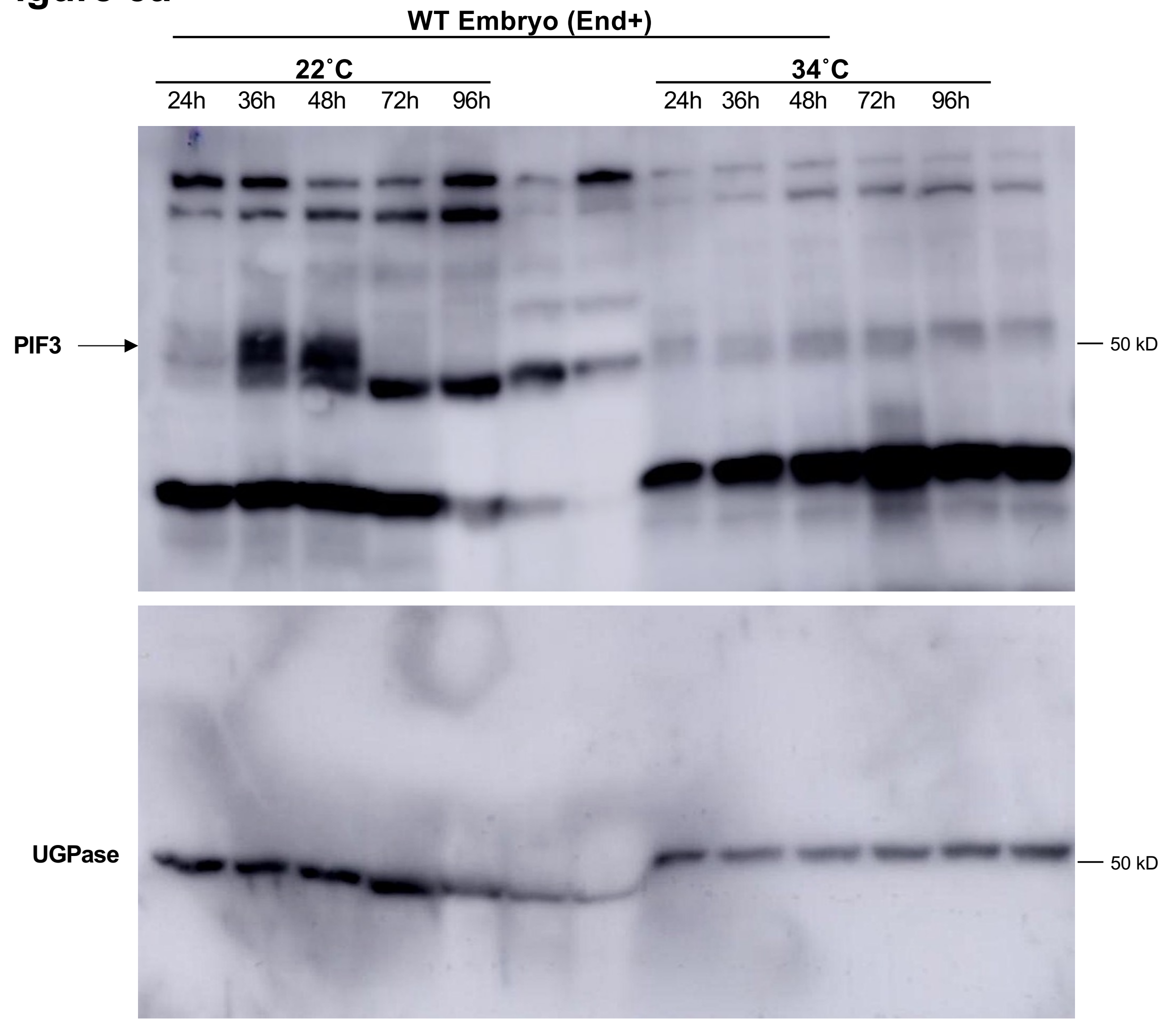

Supplementary Figure 9. Full, non-cropped protein blots presented in the main Figures and in the repetitions shown in Supplementary Figure 8.

Supplementary Figure 9

Repetition for Figure 5a (shown in suppl. Fig. 8)

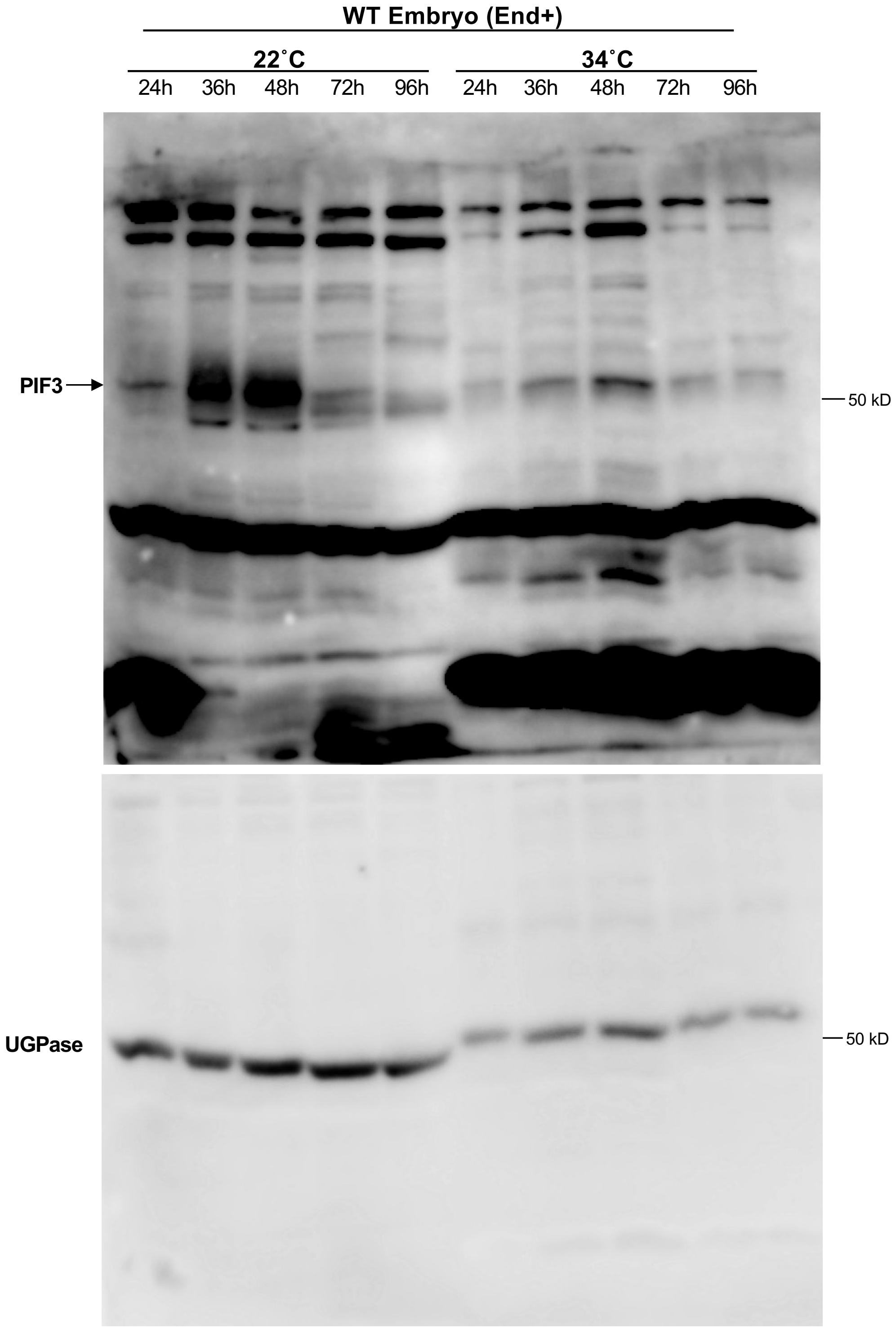

Supplementary Figure 9. Full, non-cropped protein blots presented in the main Figures and in the repetitions shown in Supplementary Figure 8.

Supplementary Figure 9

Figure 5a

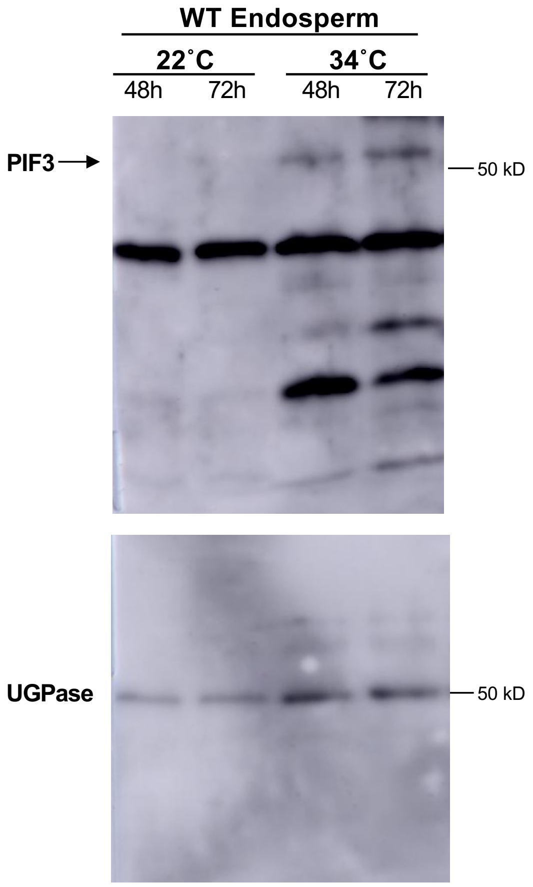

Repetition for Figure 5a (shown in suppl. Fig. 8)

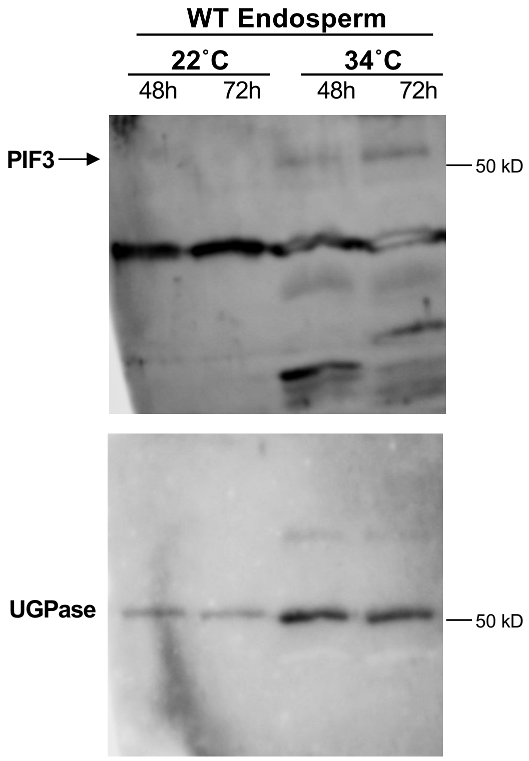

PIF3 antibody control

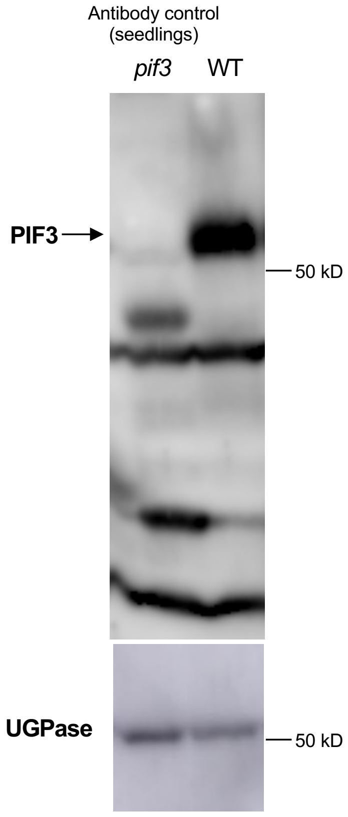

Repetition for PIF3 antibody control

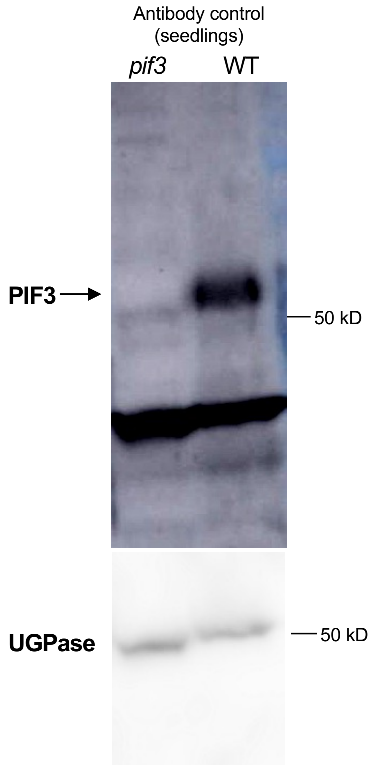

Supplementary Figure 9. Full, non-cropped protein blots presented in the main Figures and in the repetitions shown in Supplementary Figure 8.

Supplementary Figure 9

Repetition for Figure 5a (early time-points, shown in suppl. Fig. 8)

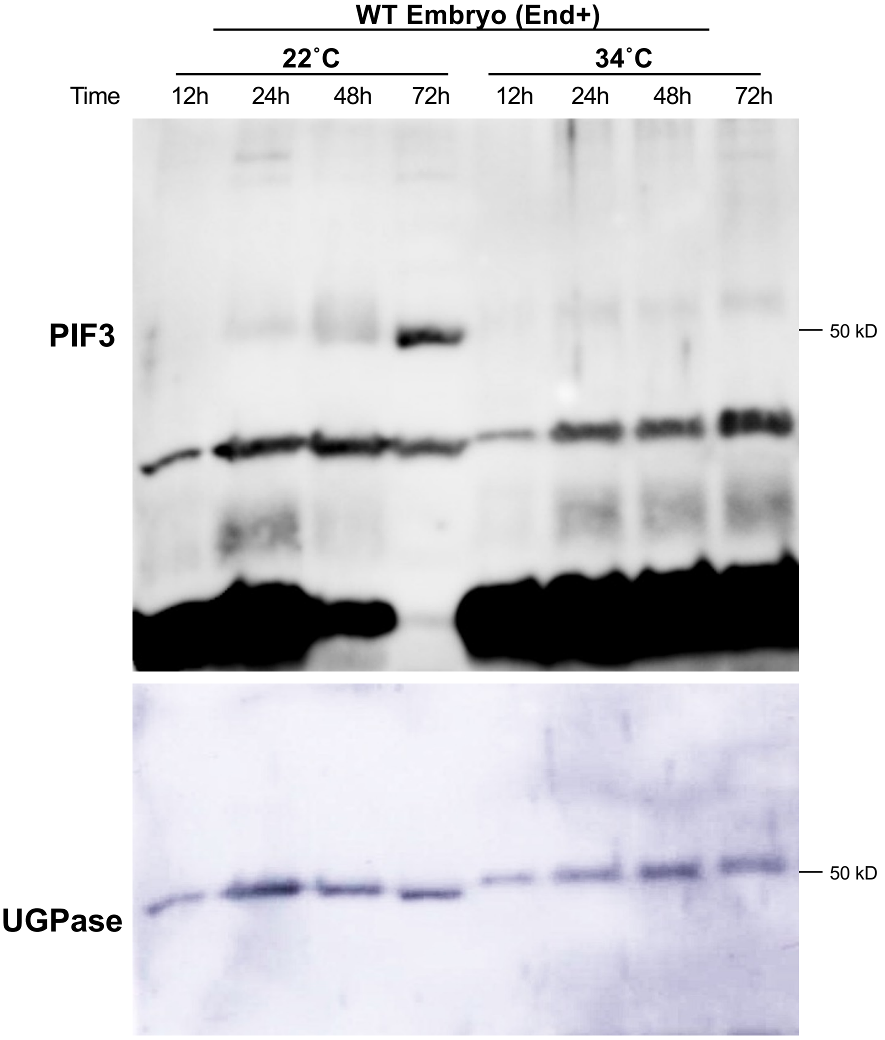

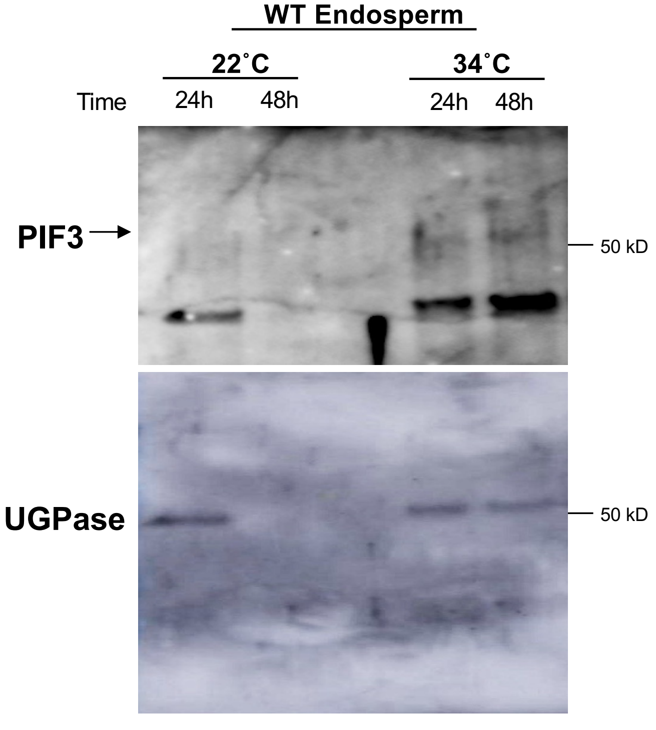

Supplementary Figure 9. Full, non-cropped protein blots presented in the main Figures and in the repetitions shown in Supplementary Figure 8.

Supplementary Figure 9

Figure 5c

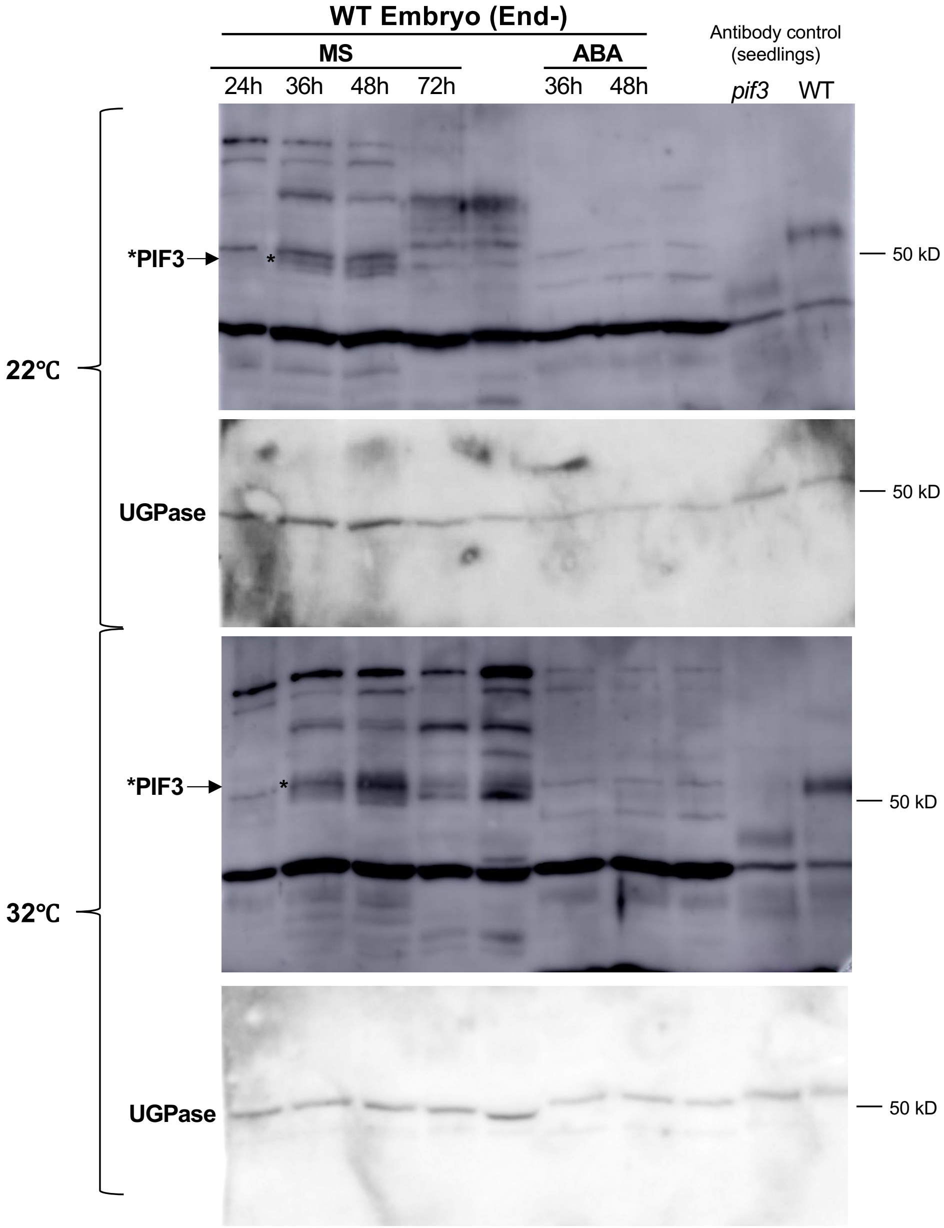

Supplementary Figure 9. Full, non-cropped protein blots presented in the main Figures and in the repetitions shown in Supplementary Figure 8.

Supplementary Figure 9

Repetition for Figure 5c (shown in suppl. Fig. 8)

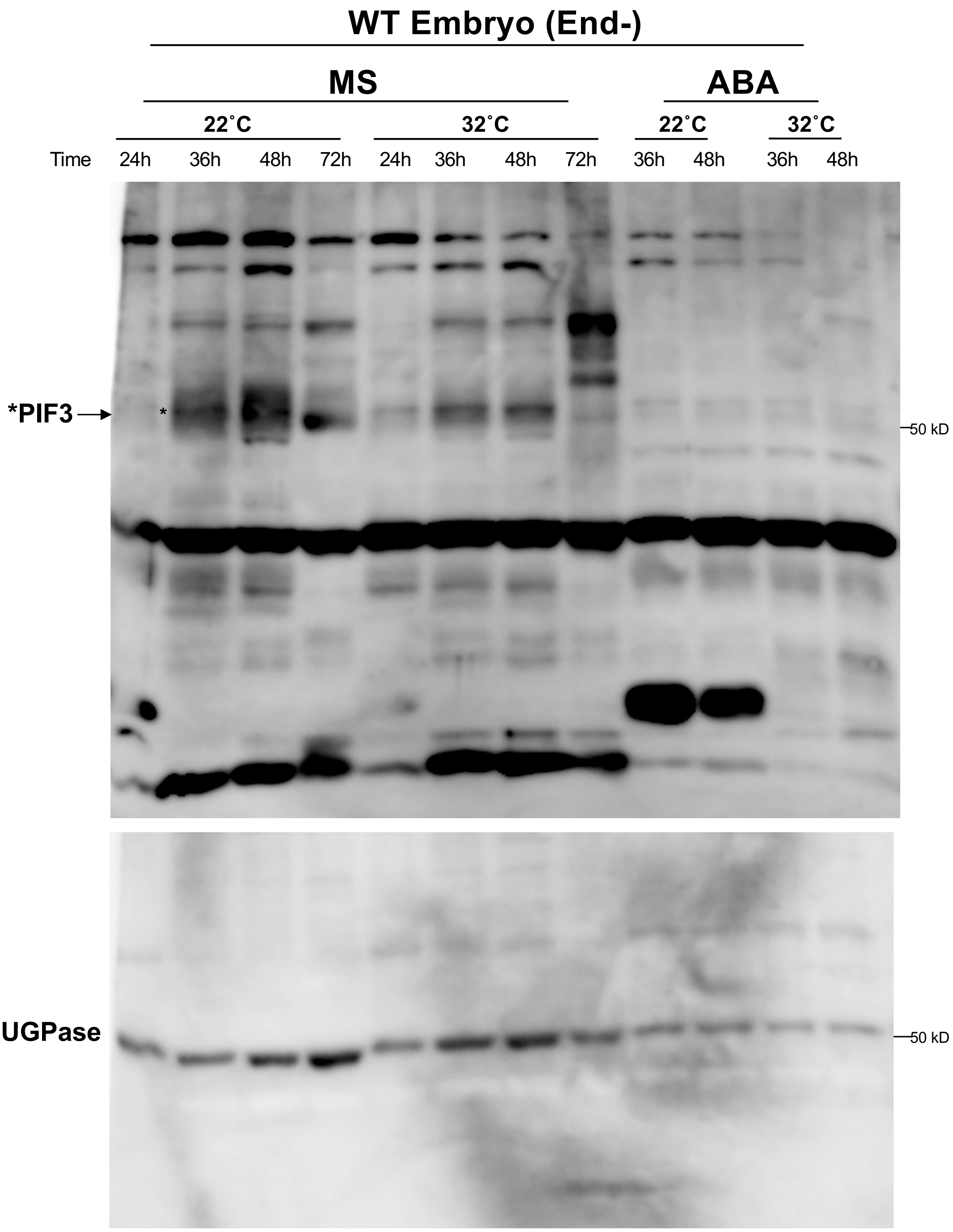

Supplementary Figure 9. Full, non-cropped protein blots presented in the main Figures and in the repetitions shown in Supplementary Figure 8.

Supplementary Figure 9

Repetition for Figure 5c (using 34˚C, shown in suppl. Fig. 8)

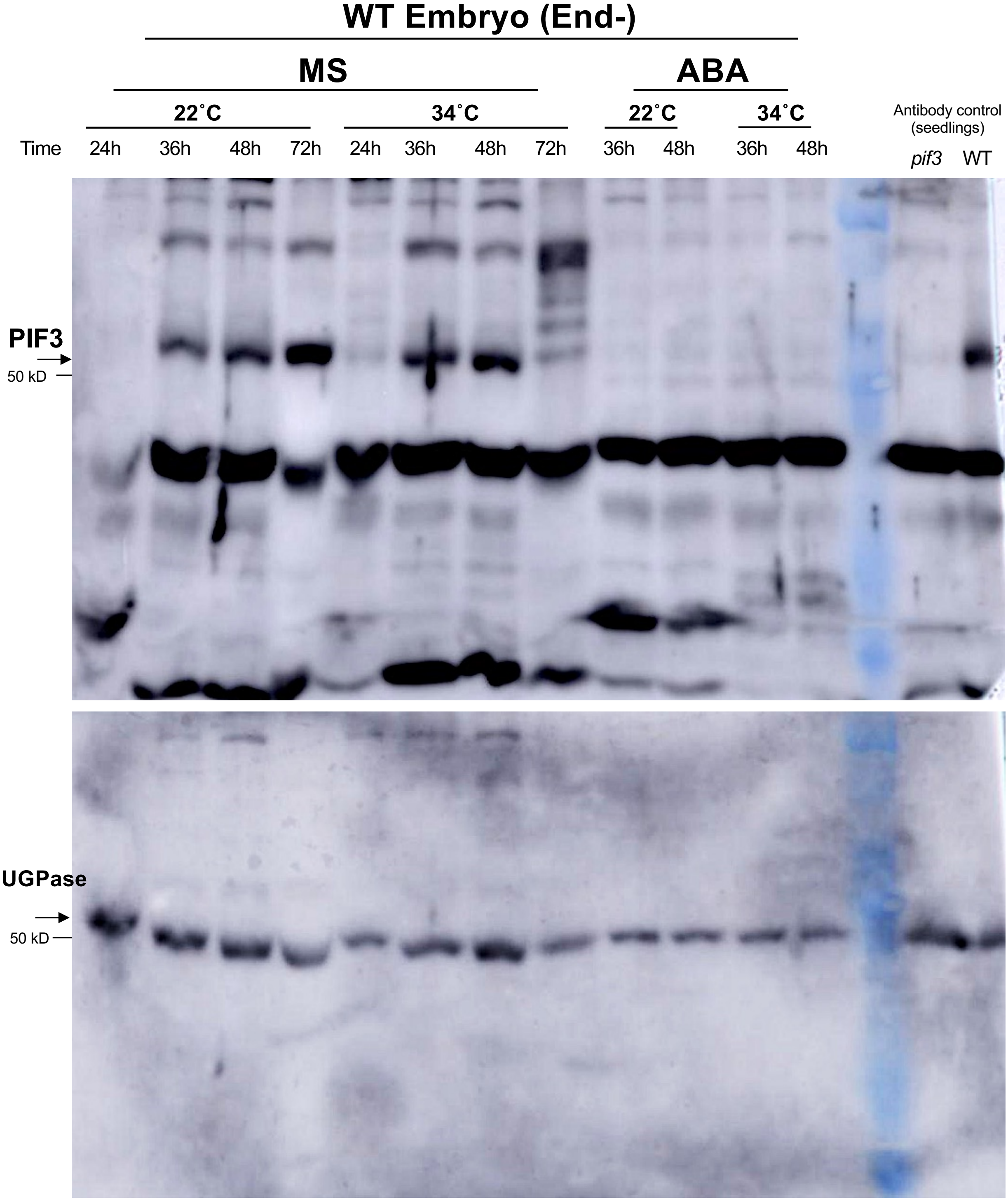

Supplementary Figure 9. Full, non-cropped protein blots presented in the main Figures and in the repetitions shown in Supplementary Figure 8.

Supplementary Figure 9

Figure 5d

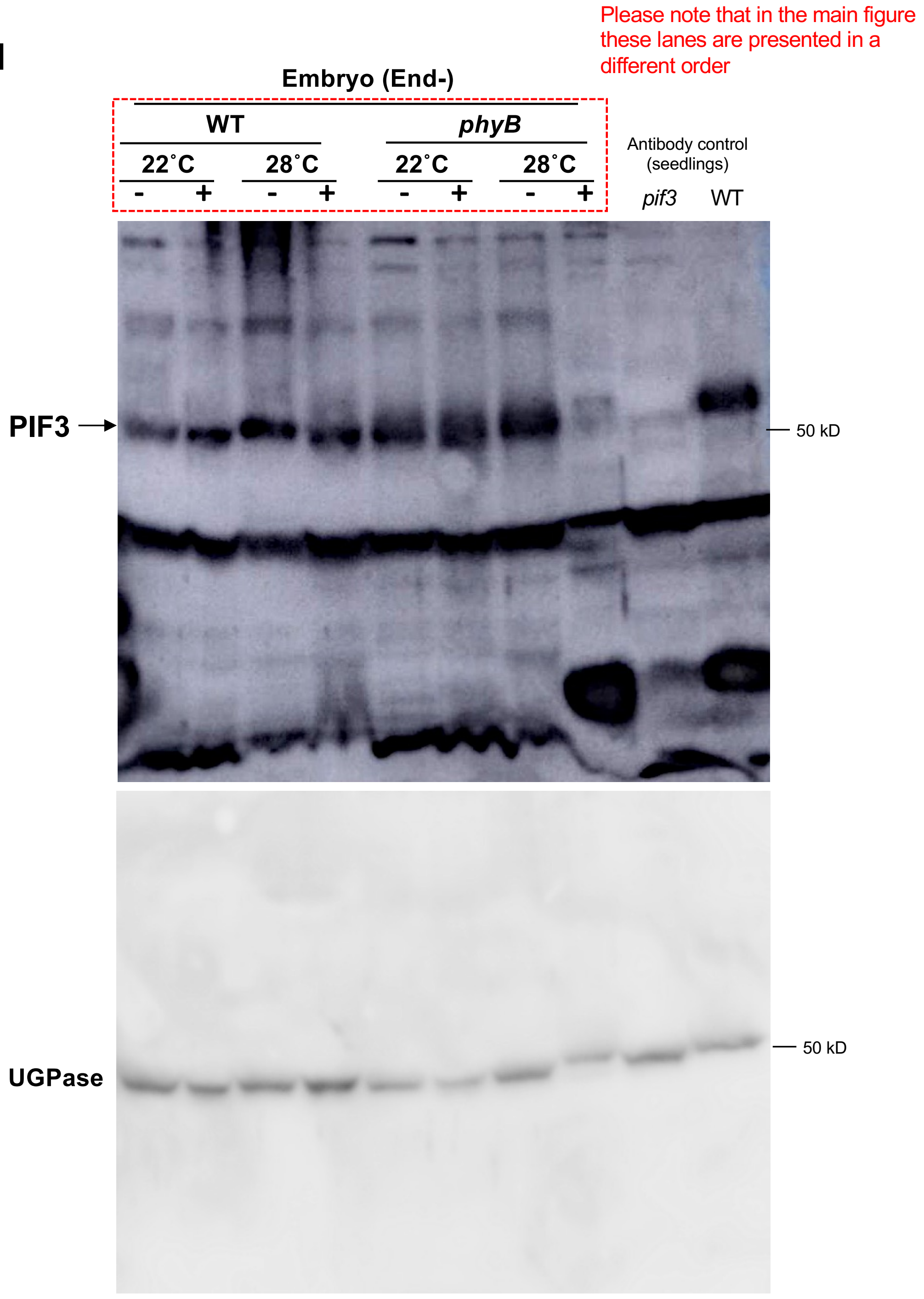

Supplementary Figure 9. Full, non-cropped protein blots presented in the main Figures and in the repetitions shown in Supplementary Figure 8.

Repetition for Figure 5d (shown in suppl. Fig. 8)

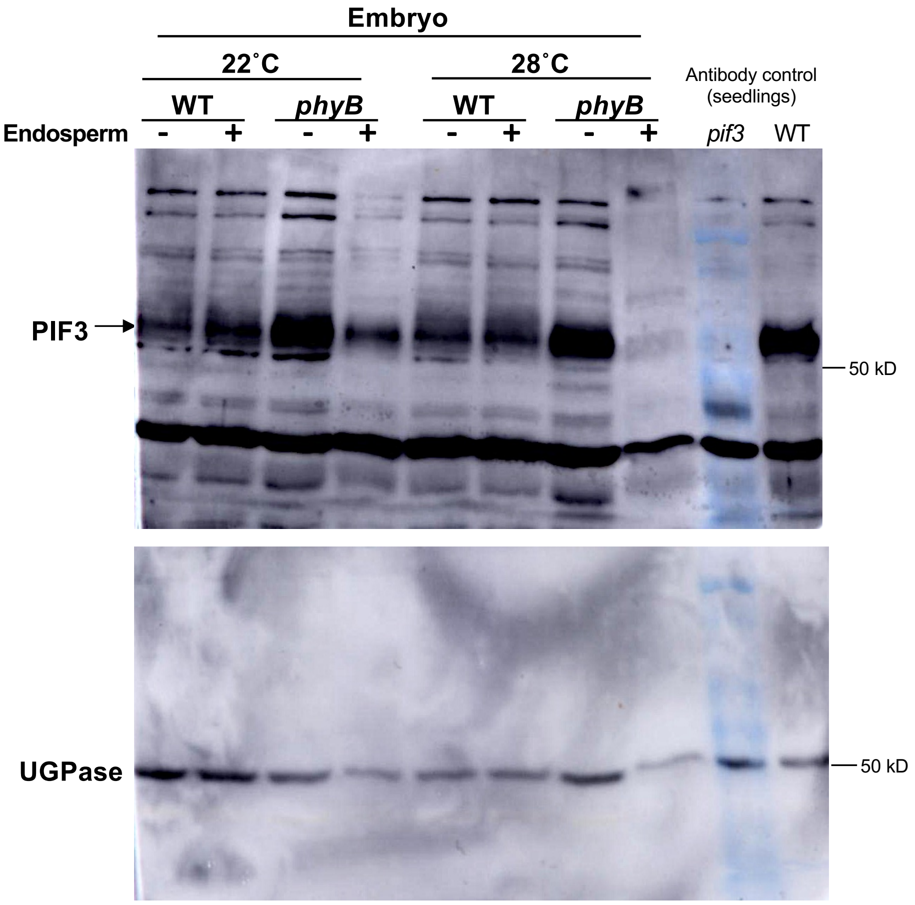

Supplementary Figure 9. Full, non-cropped protein blots presented in the main Figures and in the repetitions shown in Supplementary Figure 8.

Supplementary 1c

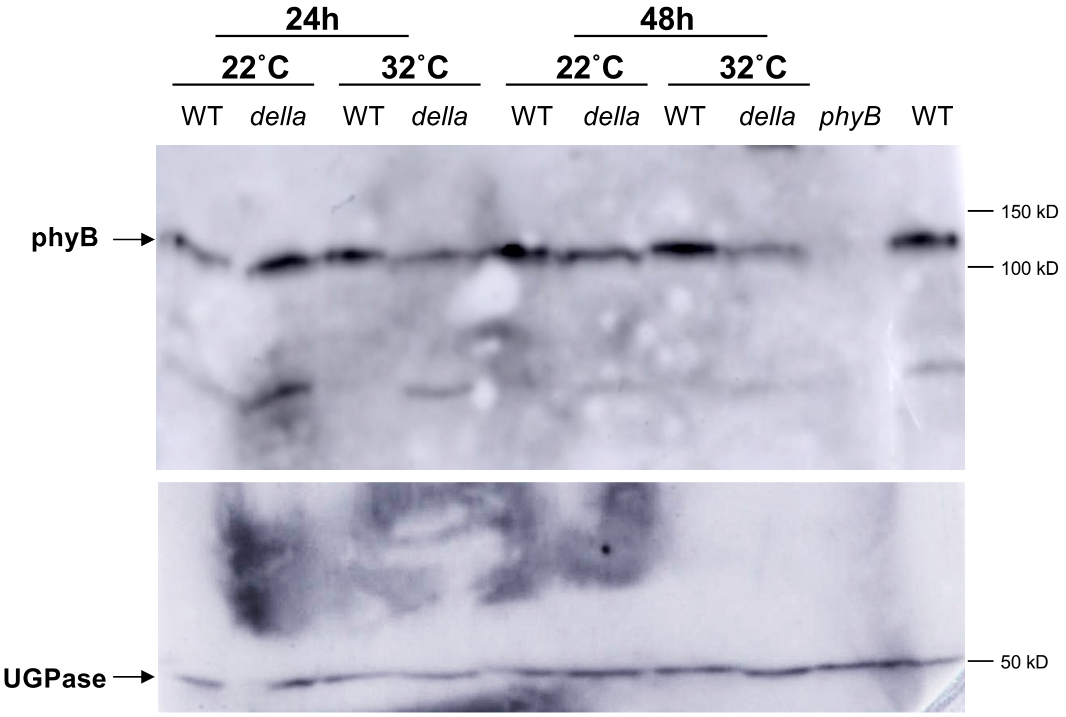

Supplementary Figure 9. Full, non-cropped protein blots presented in the main Figures and in the repetitions shown in Supplementary Figure 8.

Supplementary 7

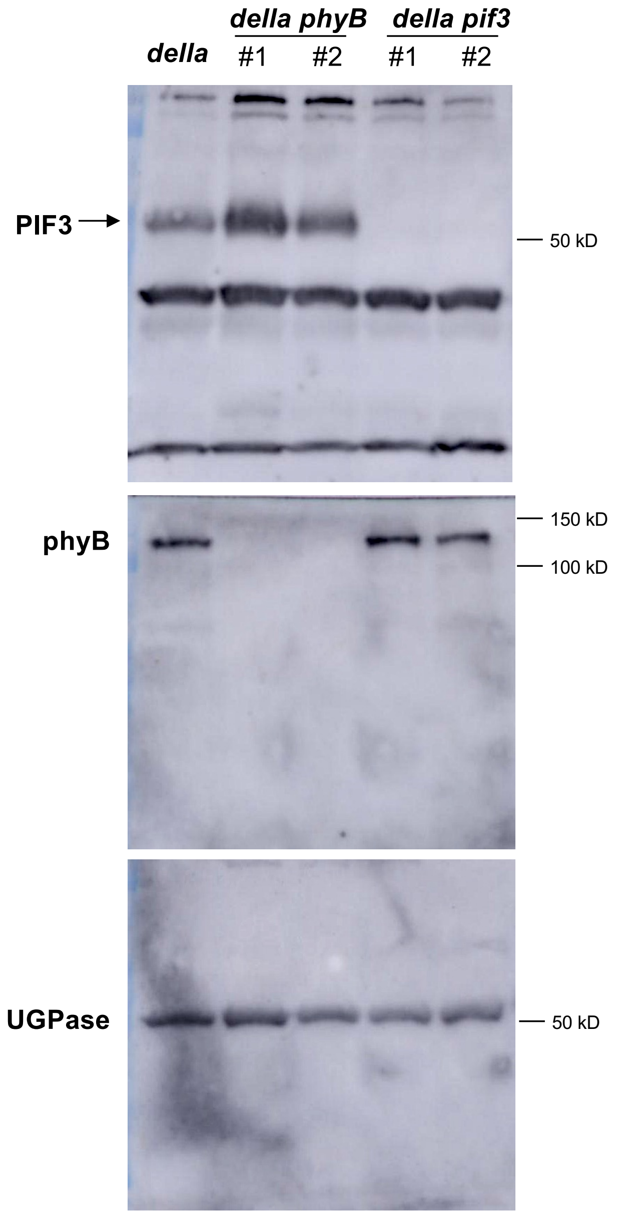

Supplementary Figure 9. Full, non-cropped protein blots presented in the main Figures and in the repetitions shown in Supplementary Figure 8.
