# Supplementary material for: The Arabidopsis endosperm is a temperature-sensing tissue that implements seed thermoinhibition through phyB
Source: Nat Commun. 2023 Mar 7;14:1202. doi: 10.1038/s41467-023-36903-4 (PMC9992654; doi:10.1038/s41467-023-36903-4)
Supplement: Supplementary file 3 — Description of Additional Supplementary Files [file 41467_2023_36903_MOESM3_ESM.pdf]

Description of Additional Supplementary Files:

Supplementary Data 1: Transcriptome of WT and pif3 endosperm and embryo from seeds imbibed for 24h and 48h at 30˚C
